# The formyl peptide receptor agonist FPRa14 induces differentiation of Neuro2a mouse neuroblastoma cells into multiple distinct morphologies which can be specifically inhibited with FPR antagonists and FPR knockdown using siRNA

**DOI:** 10.1371/journal.pone.0217815

**Published:** 2019-06-06

**Authors:** Peter J. G. Cussell, Michael S. Howe, Thomas A. Illingworth, Margarita Gomez Escalada, Nathaniel G. N. Milton, Andrew W. J. Paterson

**Affiliations:** School of Clinical and Applied Sciences, Leeds Beckett University, Leeds, United Kingdom; Universite de Rouen, FRANCE

## Abstract

The N-formyl peptide receptors (FPRs) have been identified within neuronal tissues and may serve as yet undetermined functions within the nervous system. The FPRs have been implicated in the progression and invasiveness of neuroblastoma and other cancers. In this study the effects of the synthetic FPR agonist FPRa14, FPR antagonists and FPR knockdown using siRNA on mouse neuroblastoma neuro2a (N2a) cell differentiation plus toxicity were examined. The FPRa14 (1–10μM) was found to induce a significant dose-dependent differentiation response in mouse neuroblastoma N2a cells. Interestingly, three distinct differentiated morphologies were observed, with two non-archetypal forms observed at the higher FPRa14 concentrations. These three forms were also observed in the human neuroblastoma cell-lines IMR-32 and SH-SY5Y when exposed to 100μM FPRa14. In N2a cells combined knockdown of FPR1 and FPR2 using siRNA inhibited the differentiation response to FPRa14, suggesting involvement of both receptor subtypes. Pre-incubating N2a cultures with the FPR1 antagonists Boc-MLF and cyclosporin H significantly reduced FPRa14-induced differentiation to near baseline levels. Meanwhile, the FPR2 antagonist WRW4 had no significant effect on FPRa14-induced N2a differentiation. These results suggest that the N2a differentiation response observed has an FPR1-dependent component. Toxicity of FPRa14 was only observed at higher concentrations. All three antagonists used blocked FPRa14-induced toxicity, whilst only siRNA knockdown of FPR2 reduced toxicity. This suggests that the toxicity and differentiation involve different mechanisms. The demonstration of neuronal differentiation mediated via FPRs in this study represents a significant finding and suggests a role for FPRs in the CNS. This finding could potentially lead to novel therapies for a range of neurological conditions including neuroblastoma, Alzheimer’s disease, Parkinson’s disease and neuropathic pain. Furthermore, this could represent a potential avenue for neuronal regeneration therapies.

## Introduction

The *N*-formyl peptide receptors (FPRs) are a family of G-protein-coupled receptors that were initially identified in phagocytic leukocytes, however subsequent reports have demonstrated FPRs to be expressed in multiple non-myeloid cell types and tissues throughout the body including the central nervous system (CNS) [1,2], and by virtue many novel physiological and pathophysiological roles for this receptor family have been described [3]. In humans, three separate FPR isoforms have been defined: FPR1, FPR2/ALX (formerly FPRL1) & FPR3 (formerly FPRL2), each encoded by a separate gene (*FPR1*, *FPR2* & *FPR3* respectively) [4]. FPR1 is the most commonly expressed FPR isoform in humans with high concentrations found in neuronal tissues, including the spinal cord, cerebellar system, hippocampus, as well as neurons of the sensory system, sympathetic and parasympathetic systems [2]. FPR2/ALX distribution closely mimics that of FPR1 and it is posited that these isoforms share overlapping functions in the immune system [5]. The mouse FPR (mFPR) family includes at least eight mFPR isoforms [6,7]. He *et al*. [8] studied the mouse homologs of FPR1, FPR2/ALX and FPR3 and demonstrated promiscuous binding properties and only slight differences in the responses to FPR ligands relative to the human equivalents. The putative functions of the additional mFPRs predominantly lie with olfaction, which mice rely heavily upon for communication and environmental feedback [9].

Recent studies have highlighted the involvement of FPRs in the progression of several neurological cancers. Neuroblastoma primary tumors and cell lines have been found to express FPR1; increased expression of which is correlated with high-risk disease and low survival rates [10]. Knockdown of FPR1 with shRNA delayed neuroblastoma development, while ectopic overexpression of FPR1 elicited augmented tumorigenesis in nude mice [10]. FPRs have been demonstrated to be expressed by human glioblastoma cell lines, in which it has been suggested that FPR activation exacerbates tumor malignancy through the production of angiogenic factors and the activation of epidermal growth factor [11]. Highly malignant human glioblastomas have been reported to selectively overexpress FPR1, with activation promoting cancer progression and metastasis [12]. Both formylated peptides and Annexin A1 released from necrotic glioblastoma cells have been demonstrated to promote tumor growth via activation of FPR1 [13]. Activation of FPR1 in human astrocytoma cell lines promotes motility, growth and angiogenesis. Targeting FPR1 with a specific antagonist was found to reduce astrocytoma cell motility and activation, thus prolonging the survival of tumour-bearing mice [14]. These studies demonstrate that the stimulation of FPR in neurological cancer cells leads to FPR upregulation in order to increase cell proliferation and tumor growth in an autocrine or paracrine manner. However, discrete changes in FPR modulation can lead to a range of biological responses depending on the cellular context, and as such FPR upregulation has been shown to produce stimulatory and inhibitory effects upon tumor progression depending on the cancer histotype [15].

FPRs respond to a vast number of structurally diverse ligands including endogenous peptides, bacterial derived peptides, synthetic library-derived peptides plus small non-peptide molecules and lipids. This unusual diversity of ligands has led to the classification of FPRs as pattern recognition receptors [16]. Interestingly, a number of endogenous FPR agonists have been linked with the pathogenesis of several neurodegenerative diseases. For example, FPR2/ALX has demonstrated to be a functional receptor for prion protein fragment PrP_106-126_, as well as the amyloidogenic peptides serum amyloid A and amyloid-*β*; which play important roles in the neurodegenerative activity of Alzheimer’s disease (AD) and prion diseases [17,18,19]. FPR activation is understood to mediate the pro-inflammatory activity of these amyloidogenic agonists via activation of microglia, the infiltration from the blood supply of mononuclear phagocytes exhibiting amplified adhesion, leukocyte recruitment and the production of pro-inflammatory cytokines, leading to a chronic neuroinflammatory response [20]. Conversely, FPR2/ALX has shown to mediate anti-inflammatory processes if activated by Annexin A1 leading to amyloid-*β* degradation [21]. It has also been suggested that FPRs mediate the uptake and fibril formation of amyloid-*β* in AD; transient FPR2/ALX activation in macrophages by amyloid-*β* stimulates rapid internalisation and degradation of the protein, however chronic stimulation leads to a build-up of amyloid-*β-*FPR complexes leading to the formation of fibrillar aggregates [22].

Two recent studies have demonstrated that FPR1 and FPR2 activation within mouse neural stem cells elicits proliferation and differentiation [23] via reactive oxygen species (ROS) activated pathways [24]. This is of particular interest, because if this differentiation response is replicable in other neuronal cells, it would provide evidence of a potential physiological role for FPRs within the nervous setting, and furthermore could highlight the FPR as a novel therapeutic target for neuronal regeneration. In the present study, the synthetic nonselective FPR agonist FPRa14 [25] was investigated for its ability to produce neuronal differentiation within cultured mouse neuroblastoma Neuro-2a (N2a) cells.

## Materials and methods

### Materials

Mouse and Human Neuroblastoma cells (N2a (ECACC 89121404), IMR-32 (ECACC 86041809), SH-SY5Y (ECACC 94030304)) were obtained from European Collection of Authenticated Cell Cultures (UK). Eagle’s Minimum Essential Medium high glucose (EMEM), Dulbecco’s Modified Eagle’s Medium high glucose (DMEM), all additional media components and cyclosporin H were purchased from Sigma Aldrich (UK). FPRa14, Boc-MLF and WRW4 were obtained from Tocris Bioscience (UK). *Silencer*^TM^ select siRNA duplexes for mouse Fpr1 (siRNA ID s66215), mouse Fpr2 (siRNA ID 66212) plus negative control no.1 siRNA and Lipofectamine RNAiMAX were purchased from ThermoFisher Scientific (UK). All other chemicals used were of reagent grade.

### Cell culture conditions

N2a cells were cultured in DMEM containing 2mM glutamine, 100μg/ml penicillin, 100μg/ml streptomycin, and 10% (v/v) heat inactivated fetal bovine serum (complete DMEM). IMR-32 and SH-SY5Y cells were cultured in EMEM containing 2mM glutamine, 100μg/ml penicillin, 100μg/ml streptomycin, and 10% (v/v) heat inactivated fetal bovine serum (complete EMEM). For siRNA transfections N2a cells were cultured in serum-free antibiotic-free DMEM. Cells were incubated under standard conditions of: 37°C, 5% CO_2_ in a humidified atmosphere. Cultures were passaged at regular intervals, once at 70–80% confluence.

### Cell differentiation assay

N2a, IMR32 and SHSHY-5Y cells were seeded at a density of 5000 cells/well into a 24-well culture plate in complete culture medium (400μL/well) and incubated under standard culture conditions for 24h. Complete culture medium was aspirated and wells were washed with PBS before treatment with serum-free culture medium (SFM) containing FPRa14 (0–10μM). Cells treated with SFM alone served as a negative control. The cells were then incubated under standard culture conditions for 48h in order to observe any morphological changes. For cell differentiation assays with FPR antagonists, cells were pre- incubated with Boc-MLF (0–40μM), cyclosporin H (0–40μM) or WRW4 (0–40μM) for 30min before addition of FPRa14 (8μM).

### Quantification of cell differentiation

Four random fields were examined in each well using an EVOS FL Auto 2 cell imaging system with 20x objective. Images were collected at specified timepoints up to 48h after FPRa14 administration. All quantitative morphological analyses were performed using ImageJ software (NIH). Differentiated cells were defined as any cell bearing one or more axon-like processes more than or equal to the length of the cell body radius, or that exhibited abnormal morphology traits. Morphological changes were quantitatively assessed via morphometric measurements of cell perimeter and area using tracing tool measurements on ImageJ [26,27]. Cells were also subjectively categorised by morphology type. Exclusionary criteria during image analysis were: any cell touching the image border or substantial cell clumps.

### siRNA transfections

Two siRNA duplexes targeting Fpr1, Fpr2 plus a third negative control knockdown duplex were used. Transfections were carried out using Lipofectamine RNAiMAX in antibiotic-free serum-free DMEM. Lipofectamine RNAiMAX and siRNA duplexes (10μM stock in nuclease-free water) were diluted separately in antibiotic-free serum-free DMEM in a v/v ratio of 1.5:25 and 1:50 respectively. Diluted siRNA was then added to diluted Lipofectamine in a 1:1 ratio and incubated at room temperature for 5min. siRNA-lipid complexes then added to sub-confluent N2a cells; 10μL per well for 96-well plate assays giving a final siRNA concentration of 1pM and 0.3μL Lipofectamine per well, and 50μL per well was used for 24-well plate assays giving a final siRNA concentration of 5pM and 1.5μL Lipofectamine per well. For simultaneous Fpr1 and Fpr2 knockdown, the Fpr1 and Fpr2 siRNA duplexes were added to 0.6μL Lipofectamine (96-well) at a final well concentration of 1pM, and 5pM with 3μL Lipofectamine (24-Well). Transfected cells were then incubated at 37°C, 5% CO_2_ for 48h before treatment with FPRa14 agonist [28].

### MTT cell viability assay

N2a cells were seeded at a density of 20,000 cells/well into a flat-bottom 96-well culture plate in complete DMEM (100μL/well) and incubated under standard culture conditions for 24h. N2a cells were then exposed to SFM containing FPRa14 (0-10mM) and incubated for a further 24h. The MTT assay was then conducted according to Mosmann [29]. Absorbance was measured at 550nm (test) and 690nm (background). Results were expressed as percentage control MTT reduction. For antagonist experiments, cells were pretreated with SFM containing Boc- MLF, cyclosporin H or WRW4 (0–40 μM) and incubated for 30 minutes prior to agonist exposure.

### Methods of statistical analysis

All the experimental data are expressed as mean ± standard error of the mean (SEM). Statistical differences between groups were calculated by unpaired Student’s *t*-tests for individual comparisons, or ANOVA with Dunnett's *post-hoc* test for multiple comparisons. Analysis was performed on data from at least three independent experiments. P<0.05 was considered to be statistically significant between groups. Statistical analysis was performed using SPSS version 24.

## Results

### FPRa14-induced cell differentiation

**Fig 1:**
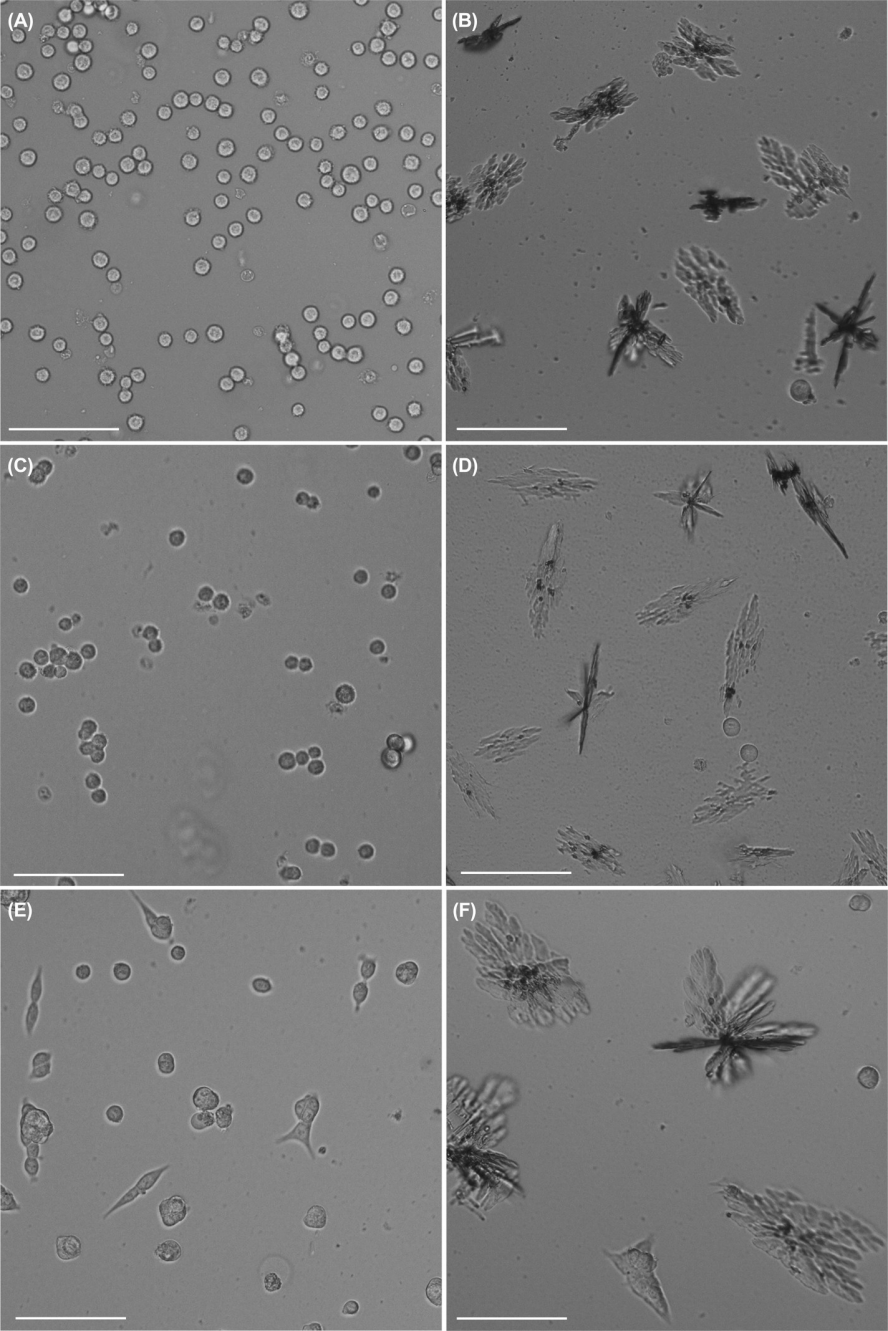
Typical phase contrast photomicrographs (200x) exhibiting **(A)** untreated N2a, **(B)** N2a treated with 10μM FPRa14, **(C)** untreated IMR-32, **(D)** IMR-32 treated with 100μM FPRa14 **(E)** untreated SH-SY5Y and **(F)** SH-SY5Y treated with 100μM FPRa14. Images were taken after 48h incubation (scale bars represent 100μm).

FPRa14 stimulated a demonstrable cellular differentiation response in neuroblastoma cell lines as shown in the typical phase-contrast microscope images displayed in **Fig 1** for N2a **(Fig 1A and 1B)**, IMR-32 **(Fig 1C and 1D)** and SH-SY5Y **(Fig 1E and 1F)**. The differentiation induced in N2a cells by FPRa14 was seen at 10μM concentrations, however in IMR-32 and SH-SY5Y a concentration of 100μM was required to produce similar effects. As a result characterization of the differentiation responses was performed on N2a cells to reduce potential for non-specific effects of both agonists and antagonists.

**Fig 2:**
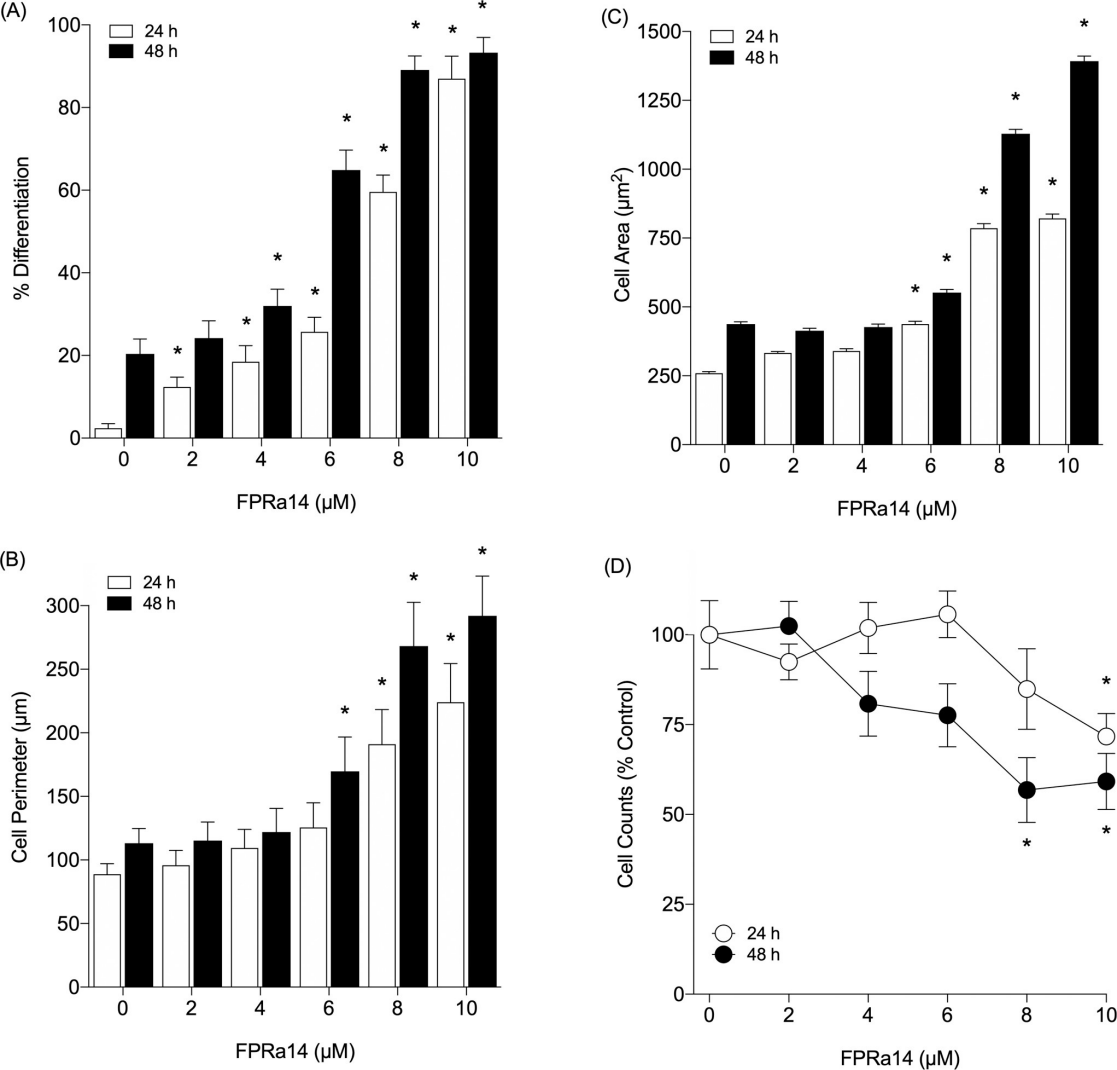
**(A)** The effect of FPRa14 (0–10μM) on **(A)** the % differentiated N2a cells, **(B)** mean N2a cell perimeter, **(C)** mean N2a cell area and **(D)** mean cell count. Serum-free medium only was used as a control. Values represent mean ± SEM, taken following 24h and 48h incubation with FPRa14. Statistical analysis was performed via one-way ANOVA with Dunnett’s *post hoc* test. *Represents statistical significance (P<0.01) relative to appropriate incubation control. Mean total cell counts are expressed as a percentage of control.

After 24h incubation, the mean proportion of differentiated cells in control cultures was 2.4% **(Fig 2A)**. FPRa14 caused a significant increase in % cell differentiation relative to SFM treated controls at concentrations of 2μM (12.4%), 4μM (18.5%), 6μM (25.7%), 8μM (59.6%) and 10μM (87.0%). After 48h, the mean proportion of differentiated cells in control cultures was 20.4%. FPRa14 elicited a significant increase in % cell differentiation versus controls at concentrations of 4μM (32.0%), 6μM (64.9%), 8μM (89.1%) and 10μM (93.3%).

Alteration of cell perimeter and cell area were selected as secondary measures of cell differentiation **(Fig 2B and 2C)**. Differentiated N2a cells generally showed cell perimeters and areas greater than their undifferentiated counterparts. After 24h, it was found that there was a significant difference in mean cell perimeter between SFM control cultures and cultures treated with FPRa14 at concentrations of 8μM and 10μM. After 48h, there was a significant difference in mean cell perimeter between controls and cultures treated with FPRa14 at concentrations of 6μM, 8μM and 10μM. Similarly cell areas were found to significantly increase in cultures treated with 6, 8 and 10μM FPRa14 relative to untreated controls after both 24 and 48h incubations. Cell numbers showed a decrease in the presence of FPRa14 which reached significance at 10μM when incubated for 24h **(Fig 2D)**. Following 48h incubation with FPRa14 decreases in cell number were observed at concentrations of 8μM and above.

**Fig 3:**
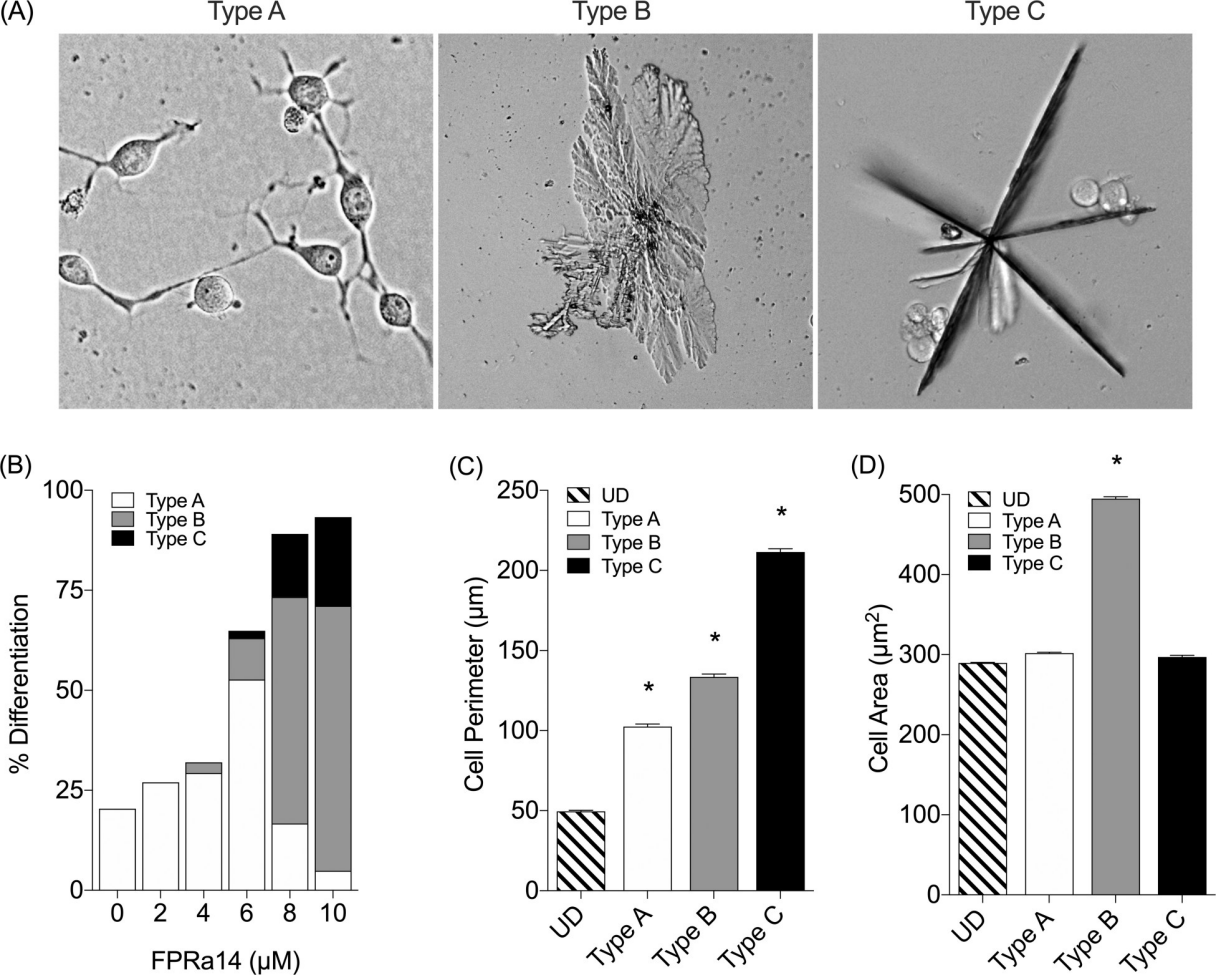
**(A)** Key highlighting examples of the three classes of differentiated cell morphology observed following incubation with FPRa14 (2–10μM). Images were taken 48h incubation with FPRa14. **(B)** The effect of FPRa14 (0–10μM) on differentiated cell morphology distribution following 48h incubation with FPRa14. (C) Mean perimeter values for each N2a morphology type observed in cultures treated with FPRa14 (0–10μM). (D) Mean area values for each N2a morphology type observed in cultures treated with FPRa14 (0–10μM). Statistical analysis was performed via one-way ANOVA with Dunnett’s *post hoc* test. *Represents statistical significance (P<0.01) relative to undifferentiated control (UD).

N2a cells which differentiated in response to FPRa14 exhibited three distinct cell body traits **(Fig 3A)** all of which were clearly distinct from undifferentiated cells. Some cells exhibited archetypal neurite formation and outgrowth (Type A), others formed into large ‘amoeboid’ structures (Type B), while others exhibited diminished cell bodies, but multiple large processes, forming a ‘star-like’ structure (Type C). Trypan blue dye exclusion was used to determine % viability of each of the forms observed. Following treatment with FPRa14 (2–10μM) for 48h the viabilities were: Undifferentiated = 59.5%, Type A = 64.0%, Type B = 85.5% and Type C = 67.2%. There were also some notable trends in the distribution of these morphology types **(Fig 3B)**: in control cultures, the only recorded differentiated cell morphology was Type-A. The Type-B cell bodies were only recorded in cultures treated with FPRa14 at concentrations ≥ 4μM, increasing in frequency in a dose dependent fashion. Type-C morphologies only existed in cultures treated with the FPRa14 at concentrations ≥ 6μM, once again becoming more prominent as the FPRa14 concentration increased. The perimeters and areas of each of the 4 cell morphologies were determined using ImageJ. All three differentiated morphologies showed significant increases in cell perimeter relative to undifferentiated control **(Fig 3C)** with the Type C (star-like) showing the greatest increase, whereas only the Type B form (amoeboid) showed a significantly increased cell area **(Fig 3D)**.

### Time course of FPRa14-induced cell differentiation

**Fig 4:**
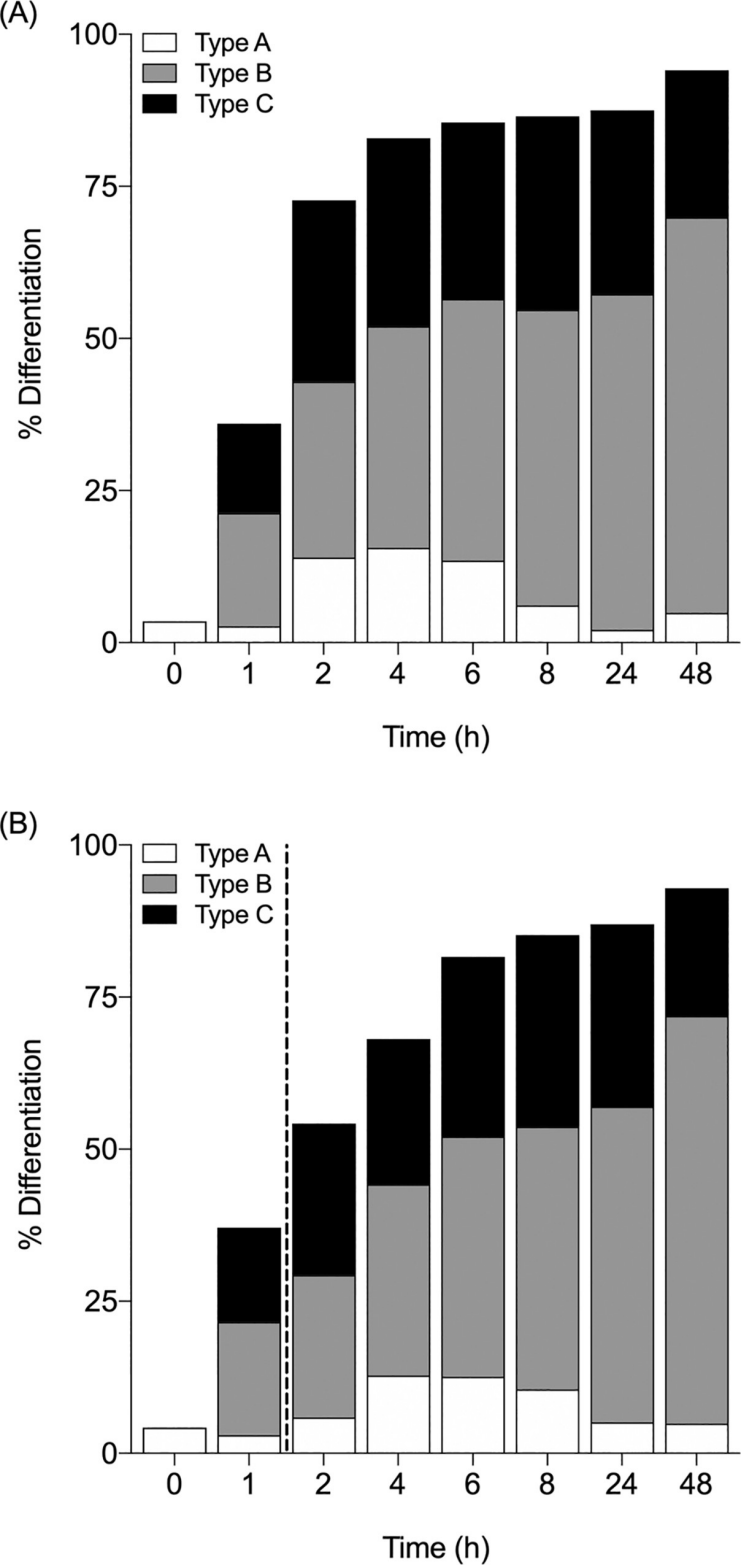
**(A)** The effect of FPRa14 (10μM) on the % of differentiated N2a cells and differentiated cell morphology distribution at time points of 0-48h following agonist administration (n = 1580). **(B)** The effect of FPRa14 (10μM) on the % of differentiated N2a cells and differentiated cell morphology distribution at time points of 0-48h when the agonist-containing media was removed after 1h incubation in the presence of FPRa14 agonist (as indicated by dotted line) and replaced with SFM (n = 1293).

In order to more closely examine the morphological changes of N2a cells during FPRa14-driven cell differentiation, additional cell differentiation assays were conducted in which images were collected at 1, 2, 4, 6, 8, 24 and 48h after treatment with FPRa14 (10μM). N2a cells were found to differentiate rapidly in response to FPRa14 **(Fig 4A)**. The proportion of differentiated cells at the point of agonist administration was 3.5%. After 1h incubation, % cell differentiation rose to 36.0% and after 2h, this value had climbed to 72.7%. Thereafter % cell differentiation stabilised (4h = 82.9%, 6h = 85.3%, 8h = 86.5%), with 24 and 48h % cell differentiation values of 86.9% and 94.0% respectively. Time-lapse data also revealed some noteworthy information on the distribution of differentiated cell phenotypes. 1h after FPRa14 administration, morphology types A, B and C **(Fig 3A)** were all observed. Type A differentiated cells underwent a transient increase in frequency, peaking at 4h where they accounted for 15.6% of the total cell population, before then diminishing in frequency, making up 2.1% of the total cell population at time-point 24h. Type A differentiated cells were once again found to increase in frequency between 24 and 48h time-points. Type B differentiated cells gradually increased in frequency with incubation time. This differentiated form accounted for 18.6% of the total cell population after 1h, and this value increased with each time-point, constituting 65.0% of the total cell population at time-point 48h. Type C differentiated cell frequency saw steady increases up to time-point 4h, where they accounted for 30.9% of the total cell population. Type C frequency then remained relatively stable until 24h where they comprised 30.2% of the total cell population.

Time-lapse cell differentiation assays were also performed in order to determine the effect of short-term exposure to FPRa14 on the N2a cell differentiation response. N2a cells were again treated with FPRa14 (10μM), however on this occasion cultures were incubated for 1h before the FPRa14-containing media was aspirated, wells washed with PBS, and fresh SFM added **(Fig 4B)**. This additional wash step reduced the proportion of differentiated cells in FPRa14-removed versus FPRa14-containing cultures during early stage differentiation. At time-point 8h FPRa14-removed N2a cultures started to achieve % cell differentiation values close to that observed in their agonist-containing counterparts. The addition of this wash step after 1h agonist exposure had little effect on the frequency and distribution of differentiated morphology phenotypes.

### Effect of siRNA targetting of Fpr1 and Fpr2 upon FPRa14-induced differentiation

In order to determine whether FPR1 and FPR2 play a functional role in the observed N2a cell responses to FPRa14, a series of siRNA knockdown experiments were conducted.

**Fig 5:**
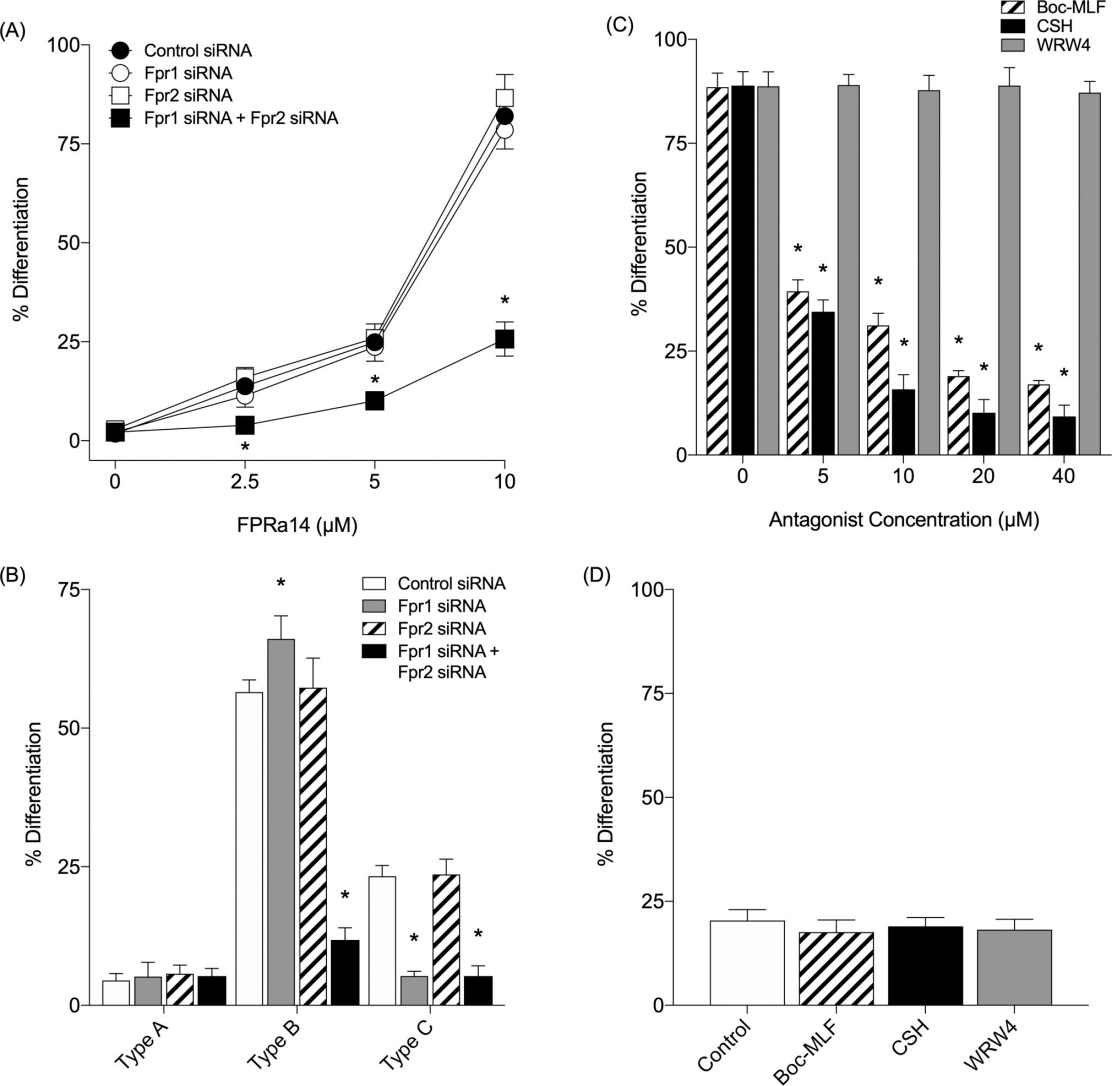
**(A)** The effect of FPRa14 (0–10μM) on the percentage differentiation of N2a cells following control siRNA, Fpr1, Fpr2, and simultaneous Fpr1 & Fpr2 siRNA treatment. **(B)** The effect of FPRa14 (10μM) on the proportion of the differentiated cell morphology types following Fpr1, Fpr2, and simultaneous Fpr1 & Fpr2 siRNA treatment. N2a cells were also transfected with a negative control siRNA duplex as a control. Values represent mean ±SEM, following 24h incubation with FPRa14. Statistical analysis was performed via one-way ANOVA with Dunnett’s *post hoc* test. *Represents statistical significance (P<0.01) relative to appropriate negative control siRNA (n = 1120). **(C)** The effect of FPRa14 (8μM) on the % of differentiated N2a cells after 30min incubation with Boc-MLF (0–40μM), cyclosporin H (0–40μM) or WRW4 (0–40μM). Serum-free medium only (SFM) was used as a negative control (n = 1121). **(D)** The effect of Boc-MLF only (40μM), cyclosporin H only (40μM), WRW4 only (40μM) and SFM on the % of differentiated N2a cells (n = 355). Values are mean ±SEM, taken after 48h of incubation with FPRa14. Statistical analysis was performed via one-way ANOVA with Dunnett’s *post-hoc* test. *Represents statistical significance (P<0.01) relative to serum-free medium control.

The effect of FPR inhibition using siRNA upon FPRa14 induced differentiation was assessed using subconfluent N2a cells 48h post transfection with siRNA duplexes targeting Fpr1, Fpr2, simultaneous targetting of Fpr1 and Fpr2 and a negative control duplex. Individual treatment with siRNA against Fpr1 and Fpr2 produced no significant change in % cell differentiation relative to negative siRNA controls when treated with FPRa14 at concentrations of 2.5–10μM **(Fig 5A)**. However when combined Fpr1 and Fpr2 siRNAs were used, there was a significant reduction in % cell differentiation relative to negative control siRNA treated N2a cells at FPRa14 at concentrations of 2.5–10μM.

Distribution of cells across the three differentiated morphology types varied with the siRNA treatments. Control siRNA treatment resulted in a similar distribution of Types A, B and C in response to FPRa14 **(Fig 5B)** to that seen in wild-type FPRa14 treated N2a cells **(Fig 3B)**. The Fpr2 siRNA had no effect on cell morphology distribution in response to FPRa14 when used in isolation. The Fpr1 siRNA resulted in a significant increase in the proportion of type B morphologies and a significant decrease in type C morphologies **(Fig 5B)** whilst not impacting the overall percentage differentiation **(Fig 5A).** Simultaneous treatment with Fpr1 and Fpr2 siRNAs resulted in significant decreases in the proportion of Type B and C morphologies **(Fig 5B)** and overall percentage differentiation **(Fig 5A)**. This suggests that FPRa14 acts via FPR1 to induce Type C morphologies, whilst the Type B morphologies can be induced by FPRa14 acting via either FPR1 or FPR2.

### Effect of FPR antagonists on FPRa14-induced differentiation

The effects of FPR antagonists on FPRa14 induced differentiation were assessed using N2a cells pre-incubated in the presence of FPR1 antagonists Boc-MLF or cyclosporin H, or the FPR2/ALX antagonist WRW4 prior to the administration of FPRa14. After 48h treatment with FPRa14, the mean proportion of differentiated cells was 88.5% in the absence of antagonist **(Fig 5C)**. Pre-incubating cells with Boc-MLF or cyclosporin H caused a significant reduction in the FPRa14 induced cell differentiation at concentrations of 5μM and above. Cyclosporin H appeared more potent in inhibition of FPRa14-induced differentiation when used at concentrations of 10μM or higher. Pre-incubation with WRW4 (up to 40μM) produced no significant change in % cell differentiation stimulated by FPRa14. Boc-MLF (40μM),cyclosporin H (40μM) and WRW4 (40μM) in the absence of FPRa14 had no significant effect on differentiation relative to serum-free medium controls **(Fig 5D)**.

### MTT cytotoxicity assay

**Fig 6:**
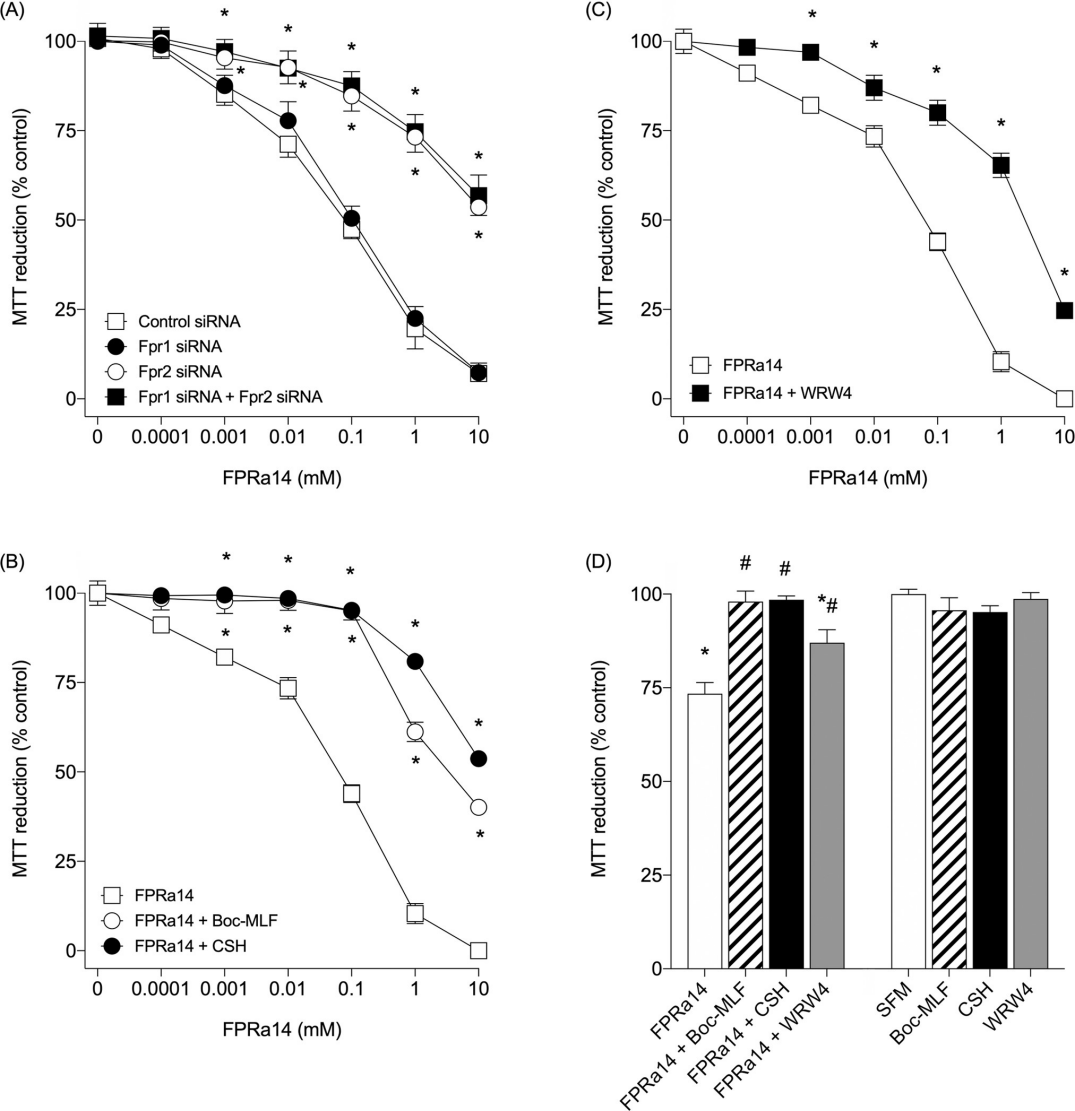
**(A)** The effect of FPRa14 (0-10mM) on % control MTT reduction in N2a cells following control siRNA, Fpr1, Fpr2, and simultaneous Fpr1 & Fpr2 siRNA treatment. Values represent mean ±SEM, following 24h incubation with FPRa14. Statistical analysis was performed via one-way ANOVA with Dunnett’s *post-hoc* test. *Represents statistical significance (P<0.01) relative to negative control siRNA plus FPRa14. **(B)** The effect of FPRa14 (0-10mM) alone, FPRa14 (0-10mM) following 30min pre-incubation with Boc-MLF (40μM) or cyclosporin H (40μM) on N2a % control MTT reduction. **(C)** The effect of FPRa14 (0-10mM) alone and FPRa14 (0-10mM) following 30min pre-incubation with WRW4 (40μM) on N2a % control MTT reduction. Values are mean ±SEM from six repeats. Statistical analysis was performed via one-way ANOVA with Dunnett’s *post-hoc* test. *Represents statistical significance (P<0.01) relative to positive FPRa14 control. **(D)** The effect of FPRa14 (10μM) alone or serum free media and FPRa14 (10μM) or serum free media following 30min pre-incubation with Boc-MLF (40μM), cyclosporin H (40μM) or WRW4 (40μM) on N2a % control MTT reduction. Values are mean ±SEM from six repeats. Statistical analysis was performed via one-way ANOVA with Dunnett’s *post hoc* test. *Represents statistical significance (P<0.01) relative to serum free media, # represents statistical significance (P<0.01) relative to FPRa14 (10μM).

FPRa14 stimulated a dose dependent toxicity as assessed using MTT assay in control siRNA treated cells. This toxicity was unaffected by Fpr1 siRNA treatment, but was attenuated in the presence of Fpr2 siRNA **(Fig 6A)**. FPRa14 stimulated a significant dose-dependent inhibition of MTT reduction **(Fig 6B)**, in a similar maner to FPRa14 with control siRNA. In the presence of FPR1 **(Fig 6B)** and FPR2 **(Fig 6C)** antagonists there was a significant attenuation of this toxicity. The highest FPRa14 concentration used in cell differentiation assays (10μM) stimulated a significant toxicity that was also reversed by FPR1 and 2 antagonists **(Fig 6D)**.

## Discussion

The synthetic FPR agonist FPRa14 stimulated clear dose-dependent increases in N2a cell differentiation. Similar differentiation effects were observed in IMR-32 and SH-SY5Y cells, but required a ten-fold higher concentration of FPRa14 to induce these effects **(Fig 1)**. The differentiation responses were characterized by measurement of percentage differentiation, cell perimeter measurements and cell area measurements **(Fig 2)**. The use of serum free media as a vehicle for FPRa14 promoted differentiation in control experiments in agreement with published literature [30]. As such the actions of FPRa14 could be additive on an already induced differentiation. The FPRa14-induced differentiation effect in N2a cells was inhibited when cultures were pre-incubated with the FPR1 antagonists Boc-MLF and cyclosporin H [31], demonstrated by decreases in percentage cell differentiation to approximately untreated control values **(Fig 5C)**, whilst pre-incubating N2a cultures with the FPR2/ALX antagonist WRW4 had no significant effect upon FPRa14-stimulated N2a cell differentiation. Pharmacologically these results suggest that FPRa14 induces differentiation via an action on the FPR1 receptor type. Treatment with siRNAs against Fpr1 and Fpr2 only reduced the overall differentiation when used in combination, suggesting that the differentiation could be induced via either FPR1 or FPR2, and it is possible that the effects with FPR1 antagonists could be due to non-specific actions on FPR2 receptors which has been reported previously for the higher doses used in our study [16]. FPRa14 induced a number of differentiated N2a phenotypes (‘neurite outgrowth’ designated Type A, ‘amoeboid’ designated Type B and ‘star-like’ designated Type C) **(Fig 3A)** in a concentration dependent manner. The cell perimeters were significantly increased in all differentiated forms, but more so for Type C differentiation **(Fig 3C)**, whereas the cell areas were only significantly changed in Type B differentiation **(Fig 3D)**. These changes become more pronounced at increased FPRa14 concentrations, which accompany changes in distribution between the differented cell types **(Fig 3B)**.

Differentiation into Type B was inhibited by combination Fpr1 and Fpr2 siRNA treatment in agreement with the overall effects on differentiation which had suggested either FPR1 or FPR2 involvement. However the Type C form was reduced in the presence of Fpr1 siRNA suggesting that differentiation into this form is FPR1 mediated. Multiple second messenger pathways have been implicated in the actions of FPR1 and FPR2 [24,32,33]. This suggests that identifying signaling pathways involved in Type B and Type C formation may be complex due to the involvement of multiple receptor types and potentially multiple signaling pathways. One way of further characterizing this would be to combine immunohistochemical analysis of signaling protein changes plus specific neuronal markers which may aid identification and categorization of the subtypes.

Time-lapse analysis **(Fig 4)** revealed interesting insights into N2a responses to FPRa14. An immediate observation that can be made is the swift nature of FPRa14-driven differentiation, with the proportion of differentiated cells reaching 82.9% after just 4h of agonist exposure **(Fig 4A)**. Another noteworthy finding is the presence of A, B and C differentiated phenotypes in N2a cultures at both time-points of 1h and 48h post FPRa14 administration. This could suggest that each phenotype exists independently of one another, rather than a transitional differentiation from neurite outgrowth leading to ‘amoeboid’ and ‘star-like’. Removal of the agonist after 1h **(Fig 4B)**, thus removing the agonist and any secreted modulators from the extracellular environment, caused a slight delay in cell differentiation. However the removal of the extracellular stimulus had little effect on the eventual differentiated cell phenotype distribution observed. FPRs have been widely shown to undergo homologous desensitisation upon continuous stimulation by the same ligand, driven by changes in phosphorylation, internalisation and down-regulation of expression [34,35]. The *E*. *coli* -derived formyl peptide fMLF has been shown to undergo internalisation within 30 seconds of binding FPR1 [36]. FPR signaling is typically driven though G-protein interactions, however upon internalisation, it has been suggested that the occupied FPR quickly couples with the cytoskeleton within the plasma membrane [37]. In desensitised cells, FPRs are confined to domains with little access to G-proteins, but are instead exposed to cytoskeletal proteins such as actin; thereby halting G-protein related signaling and instead activating cytoskeletal transduction pathways [38]. Furthermore, when associated with the cytoskeleton, FPR has been shown to enter a “super high affinity state” characterised by an extremely low rate of dissociation of bound ligand [39]. This could explain the formation of amyloid-*β-*FPR complexes leading to the formation of fibrillar aggregates [22]. These high affinity states have also been demonstrated in other GPCR receptor types such as lectin-induced association of nerve growth factor receptors with the cytoskeleton of PC-12 cells leading to a five-fold decrease in receptor dissociation rates [40]. It is plausible that the FPRa14 ligand is internalised upon receptor activation, where FPR-FPRa14 complexes can bind to and activate certain cytoskeletal processes leading to cellular differentiation. Once associated with the N2a cytoskeleton, the rate of dissociation of FPRa14 ligand from its receptor may decrease, meaning that continuous stimulation is not necessary for cellular differentiation to continue. Our experiments with removal of the agonist containing media after 1h are unlikely to modify this type of action.

Cytotoxic effects of FPRa14 on N2a cells were characterised via MTT assay **(Fig 6)**. The dose-dependent toxicity was inhibited by FPR1 and FPR2 antagonists, however the effect was only reduced by siRNA when Fpr2 was targeted. This suggests that the toxicity response is via the FPR2 receptor and that the effects observed were due to a loss of specificity of the FPR1 antagonists at the doses used. It has been reported that Boc-MLF and cyclosporin H are specific for FPR1 [10,16] but Boc-MLF can antagonise FPR1 and FPR2/ALX at higher concentrations, whilst WRW4 is specific for FPR2/ALX [31].

The FPRa14 agonist’s ability to activate both FPR1 and FPR2/ALX [25] is much more likely to mimic the actions of naturally occurring endogenous and exogenous FPR agonists that target multiple receptor isoforms [4].

Our results suggest that both FPR1 and FPR2 are activated in N2a differentiation responses, whilst FPR2 is involved in the N2a toxicity responses observed. The siRNA experiments suggest that N2a cells endogenously expresses both FPR1 and FPR2 that, when activated via FPRa14, promotes neuronal differentiation leading to neurite outgrowth and other unique morphological responses at low micromolar concentrations. Studies in rat neural stem cells show the activation of FPR1 and FPR2/ALX leads to increased migration and differentiation [23,24] suggesting that FPR-induced differentiation may be a general feature of neuronal cells.

The involvement of a different pathway in the FPRa14 induced toxicity could play a role in some of these observed effects on differentiation, as FPRa14 was found to trigger N2a cell differentiation at concentrations of 2–10μM **(Fig 2)**, whilst it also elicits slight neurotoxicity at a concentration of 10μM **(Fig 6D)**. It can be postulated that growth factor expressional changes [11] could occur within N2a populations at sub-toxic agonist concentrations in order to increase proliferation and survival. It is important here to consider the implications of the presence of cell carcasses within the test fields. Whilst it is likely that the majority of cell material becomes detached from the culture plate following apoptosis and therefore should not be present within the photomicrographs used in these assays, there is a distinct possibility that a small proportion of cell carcasses as well as other cellular debris will remain impacting results. To negate this possibility the viability of the differentiated forms was determined using trypan blue dye exclusion and this was shown to be greater than 60% for all differentiated morphologies when cultures were exposed to the highest agonist concentrations used (10μM).

This study has demonstrated FPRa14-stimulation of both differentiation and cytotoxicity in N2a cells–interestingly, there are several possible implications of this phenomenon when the *in vivo* situation is considered. FPRs are known to be present in high concentrations within the olfactory sensory systems such as the vomeronasal organ utilised by many mammals to identify the presence of odorants and pathogens [9]. Olfactory dysfunction has been highlighted as an early clinical symptom of AD, and is linked to the neurodegeneration seen in AD [41]. A key compound in the pathogenesis of AD is amyloid-*β*, which is also a known agonist of FPR1 and FPR2/ALX [16]. Amyloid-β is also linked to other conditions including Parkinson’s disease [42]. FPR activation has demonstrated to increase the production of ROS [24], abnormally high levels of which have been detected in the brain and bloodstream of AD patients [43]. It is therefore possible that amyloid-*β* activates FPRs within nervous tissues, leading to the generation of ROS and the activation of microglia leading to chronic neuroinflammation and cell death, and thus exacerbating the progression of AD. In the same way, it is possible that activation of FPR via the prion peptide fragment PrP_106-126_, another known FPR agonist [16], may exacerbate the progression of prion disease, in which ROS generation and microglial activation are known to contribute to disease pathogenesis [44].

Increased FPR1 expression in neuroblastoma primary tumours is correlated with high-invasiveness and low patient survival rates [10], which may be influenced by the FPR-induced differentiation reported here. FPR1 is known to play a role in tumourigenicity of other cancers including glioblastoma [13] and hepatocellular carcinoma [45]. The agonist FPRa14 has been demonstrated to increase neutrophils and stimulate hepatocarcinogenesis in zebrafish [46]. Our results in combination with these suggest the potential for FPR antagonism as a therapy for neuroblastoma. Baracco *et al*. [47] found that specific FPR1 antagonism reduced the efficacy of chemotherapy in a mouse breast cancer model via an immmunosupressive action, whilst this is not in a neuronal setting it may suggest that the multiple actions of FPR could play a role *in vivo* and limit the effectiveness of FPR1 antagonism in cancer chemotherapy. It is worth noting that FPR1 activation has a cytotoxic effect in our model and therefore FPR1 antagonism could potentially block a beneficial action in a cancer setting.

This study demonstrates the ability of FPR2 activation to cause toxicity whilst combinations of FPR1 and/or FPR2 activation elicits neuronal differentiation, providing evidence for the idea that the functionality of this receptor family stretches beyond the immune and inflammatory responses. The observation of neuronal differentiation mediated via FPR in this study, plus the demonstration of FPR-mediated neural stem cell differentiation by Wang *et al*. [23] and Zhang *et al*. [24] together represent significant findings and suggest a role for FPR in the CNS. If the function of FPRs within the CNS could be further characterised, it may bring about promising advancements in the field of neuronal regeneration, and novel therapies for a range of conditions including strokes, neuropathic pain, neurological cancers and neurodegeneration.

## Supporting information

S1 Figs Exemplar images used in data analysis. (PDF)

S1 Tables Minimal dataset used in preparation of the figures. (XLSX)
