# Supplementary material for: The formyl peptide receptor agonist FPRa14 induces differentiation of Neuro2a mouse neuroblastoma cells into multiple distinct morphologies which can be specifically inhibited with FPR antagonists and FPR knockdown using siRNA
Source: PLoS One. 2019 Jun 6;14(6):e0217815. doi: 10.1371/journal.pone.0217815 (PMC6553754; doi:10.1371/journal.pone.0217815)

Exemplar Images used for analysis

Neuro2a Control

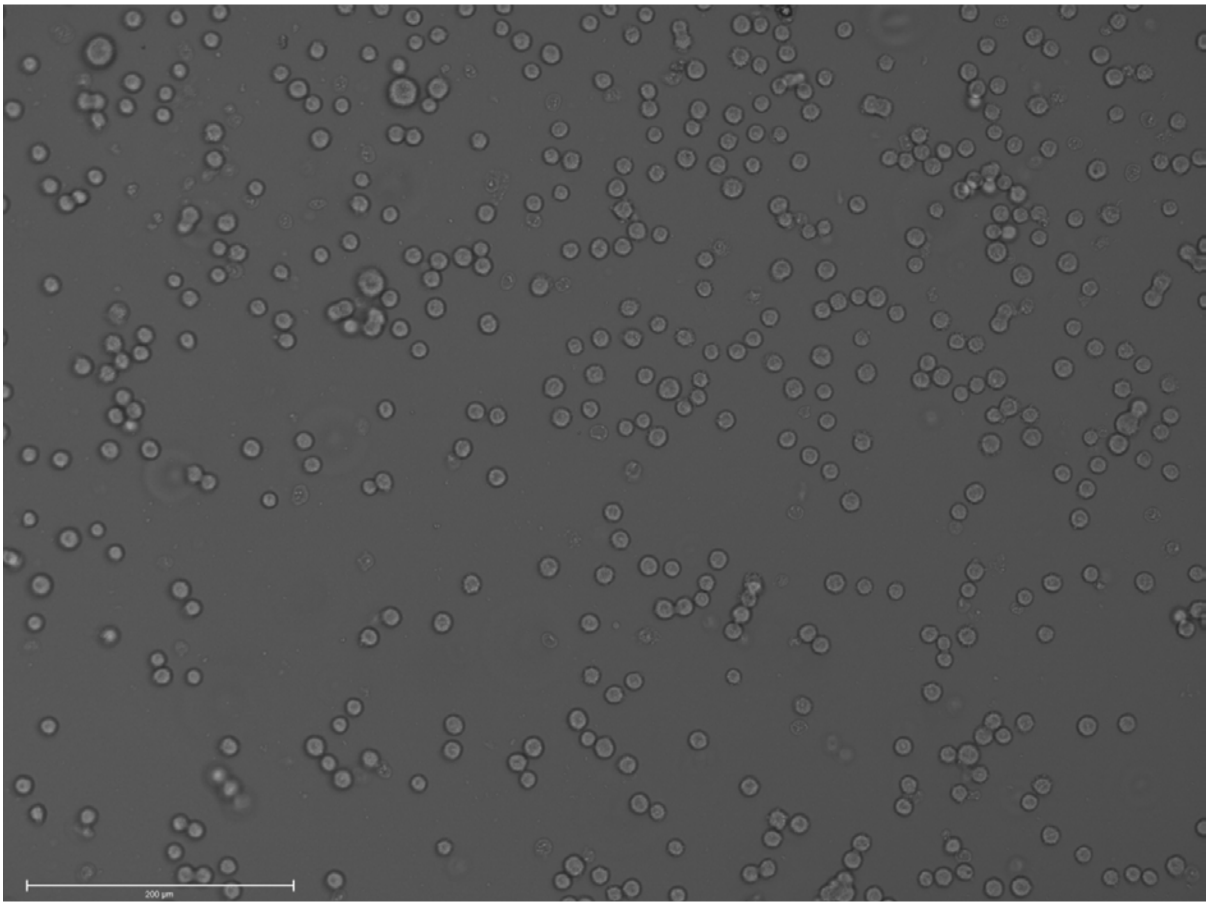

Neuro2a Control

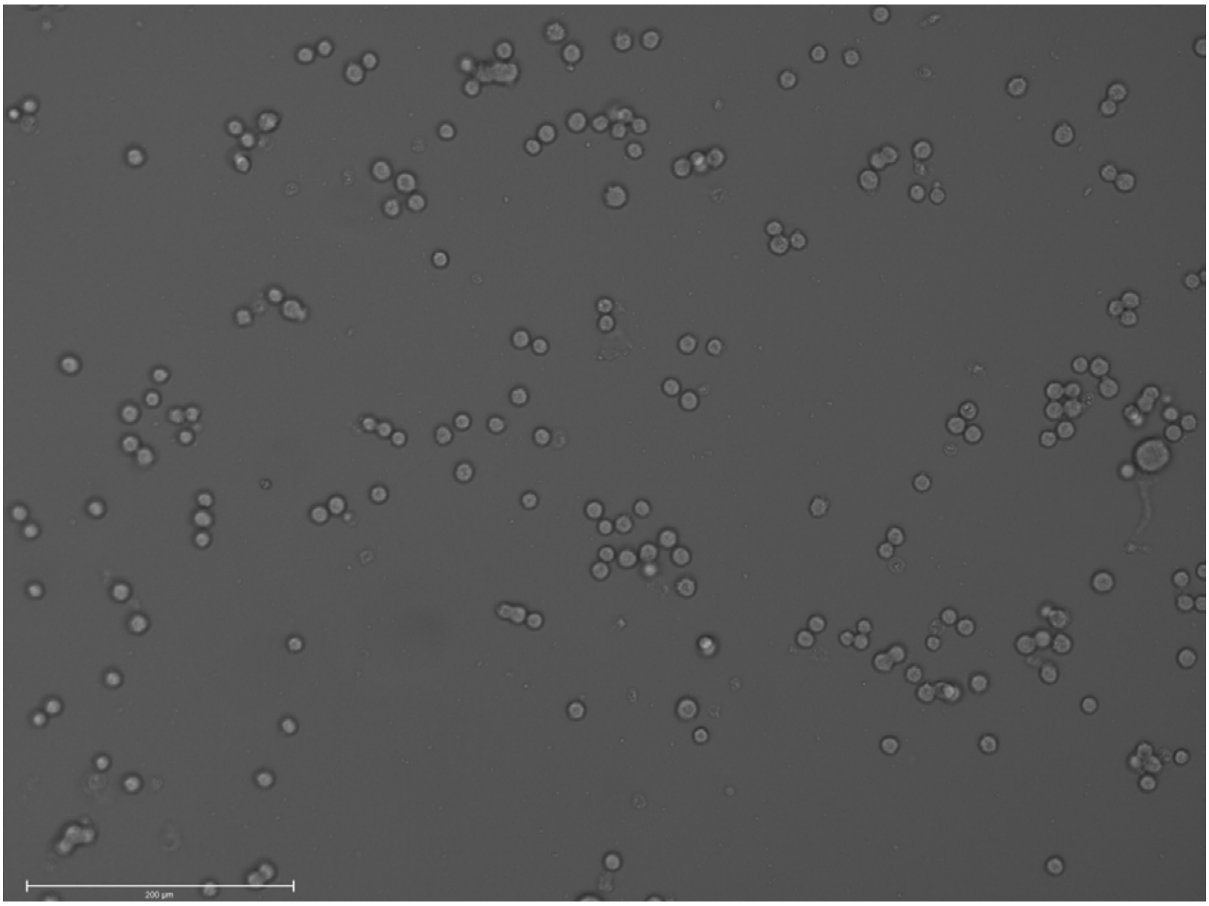

Neuro2a + 10µM FPRa14

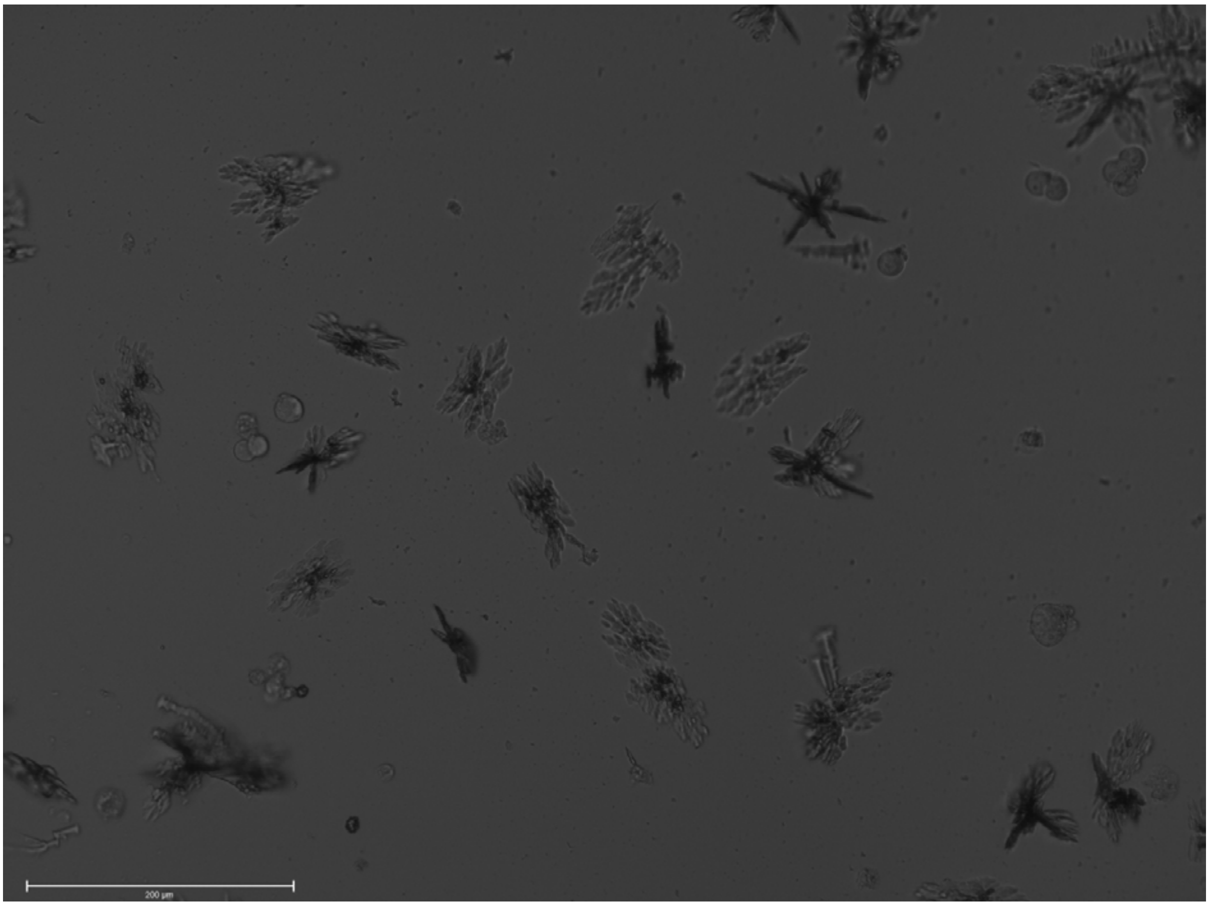

Neuro2a + 10µM FPRa14

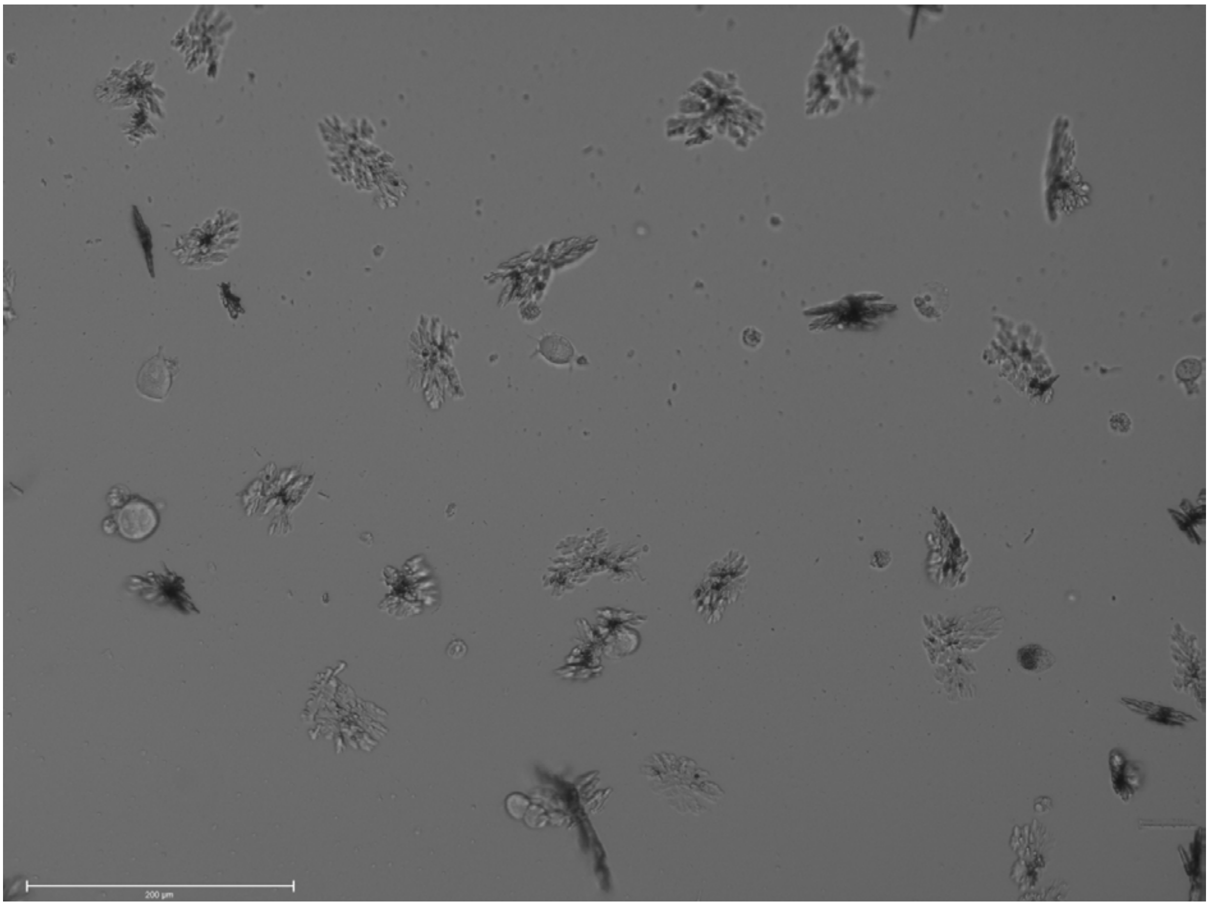

Neuro2a + 10µM FPRa14

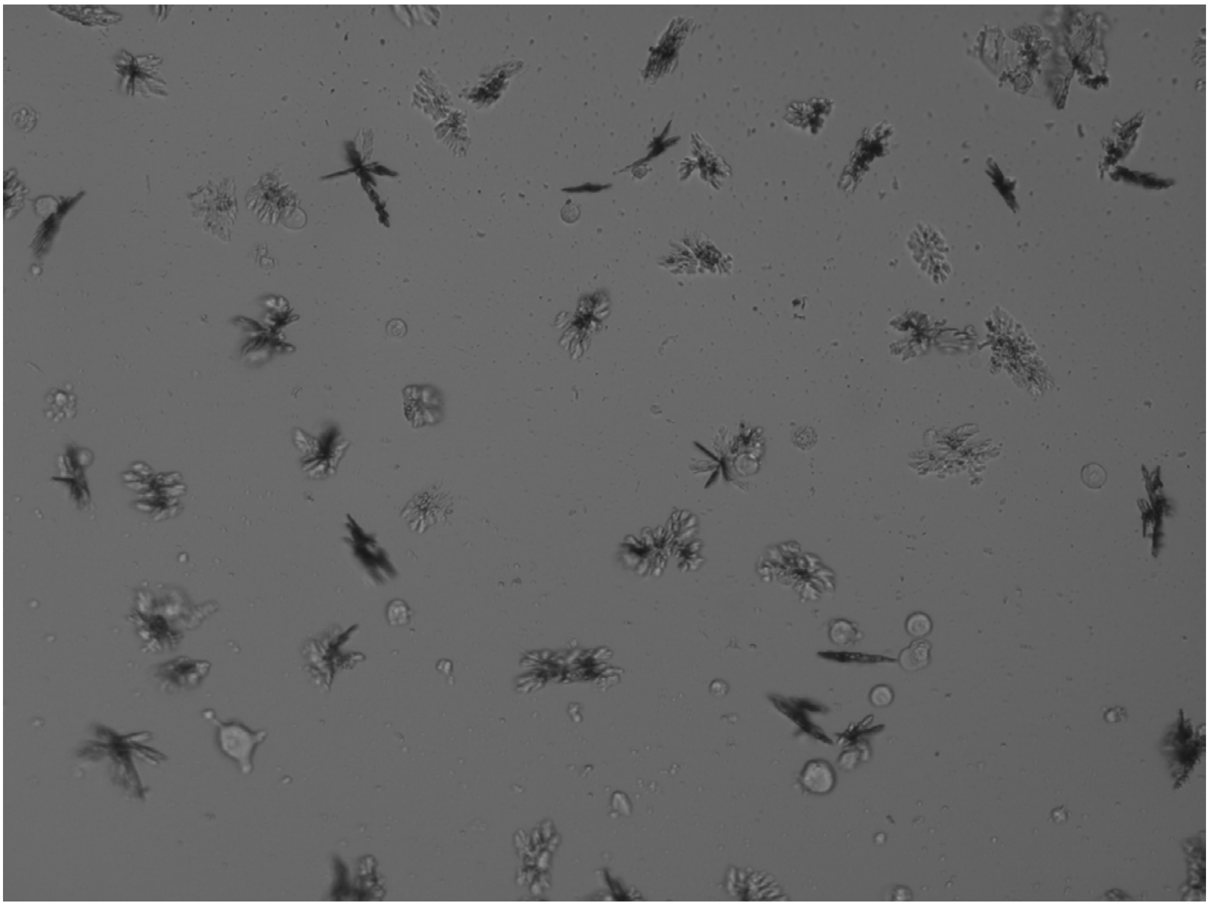

Neuro2a Type A

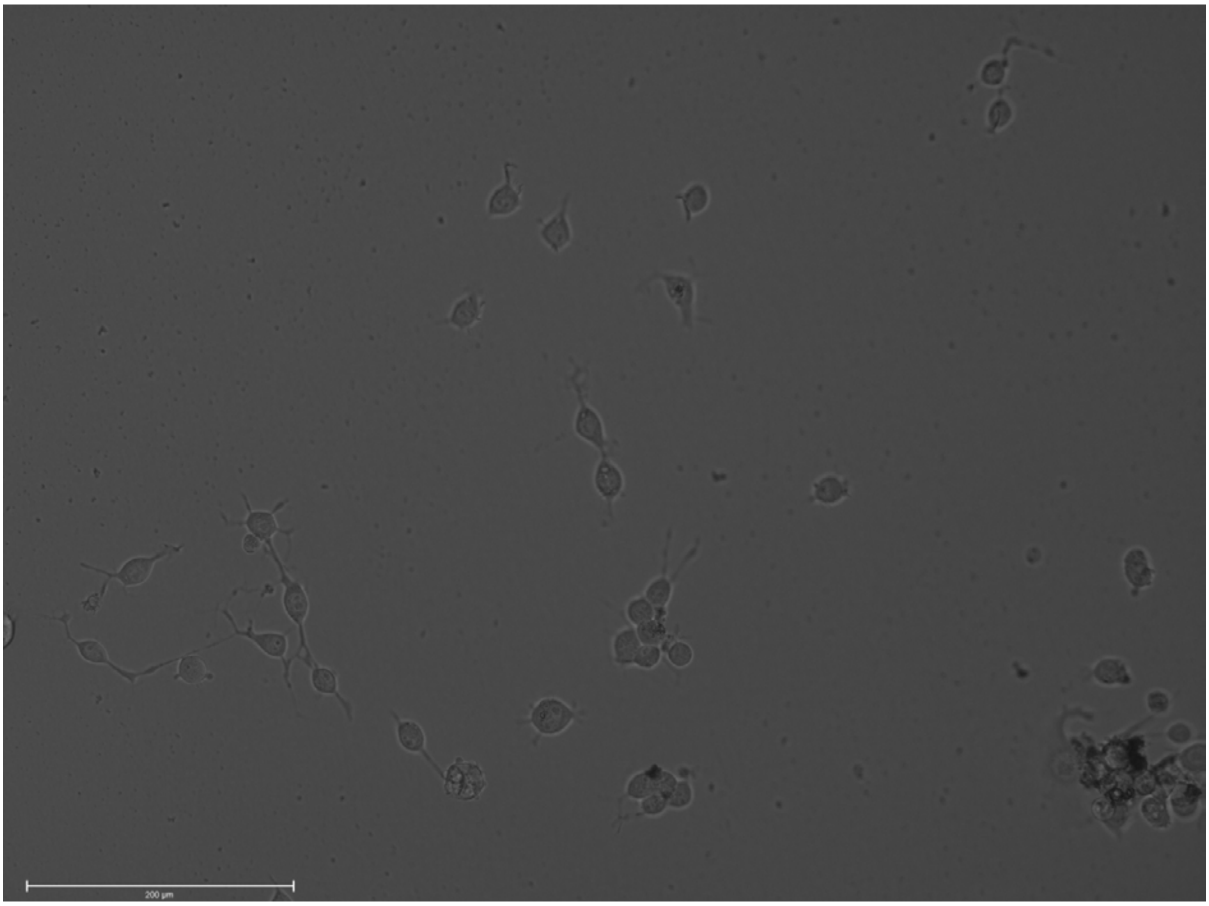

Neuro2a Type A

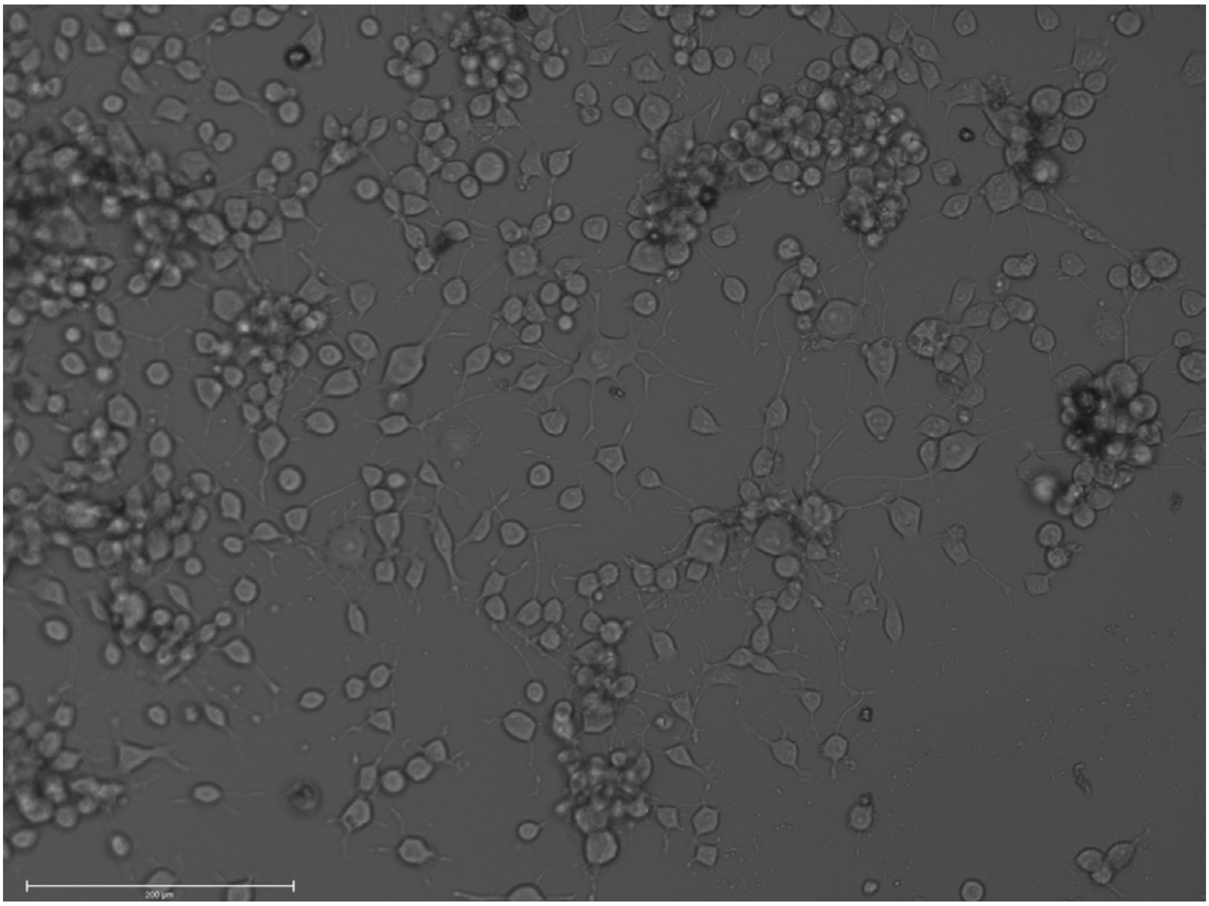

Neuro2a Type B

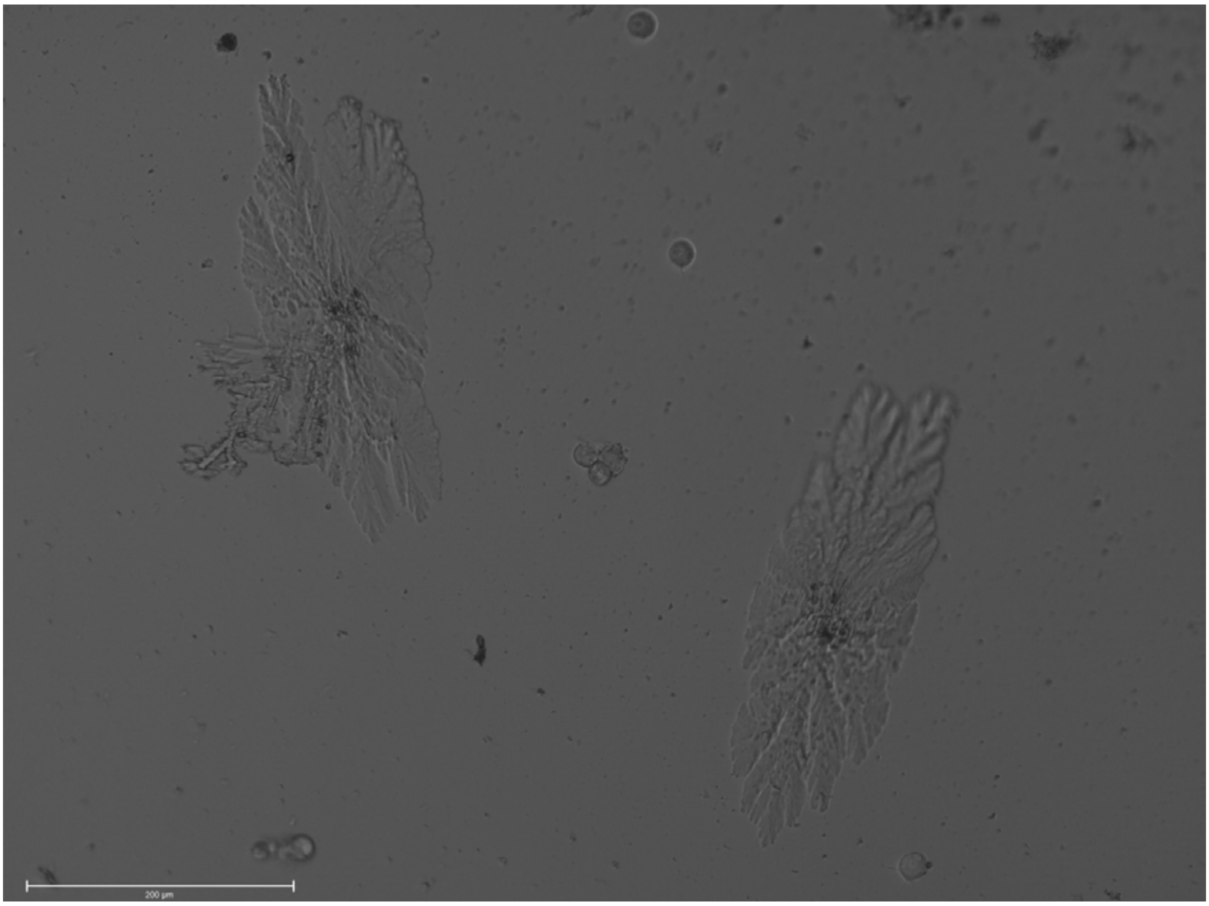

Neuro2a Type B

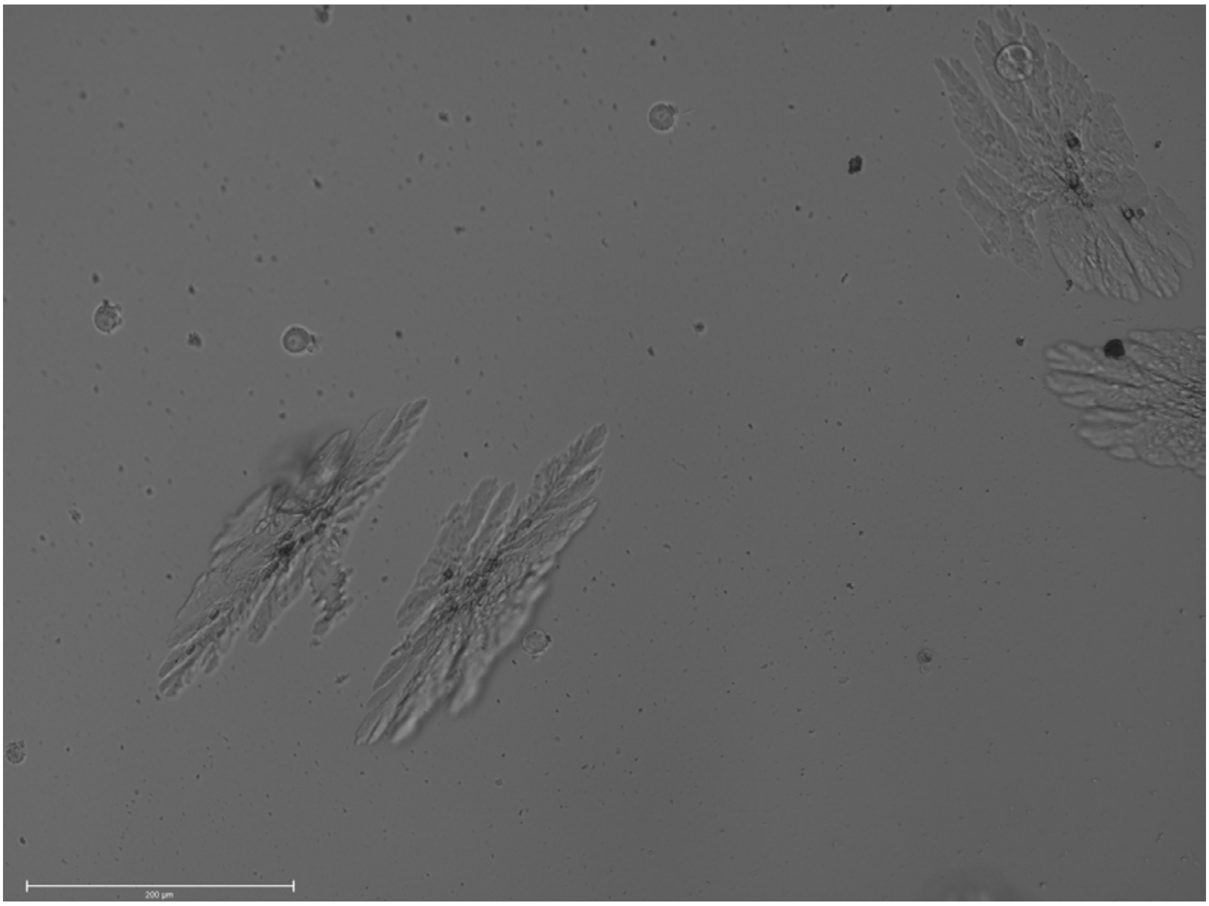

Neuro2a Type C

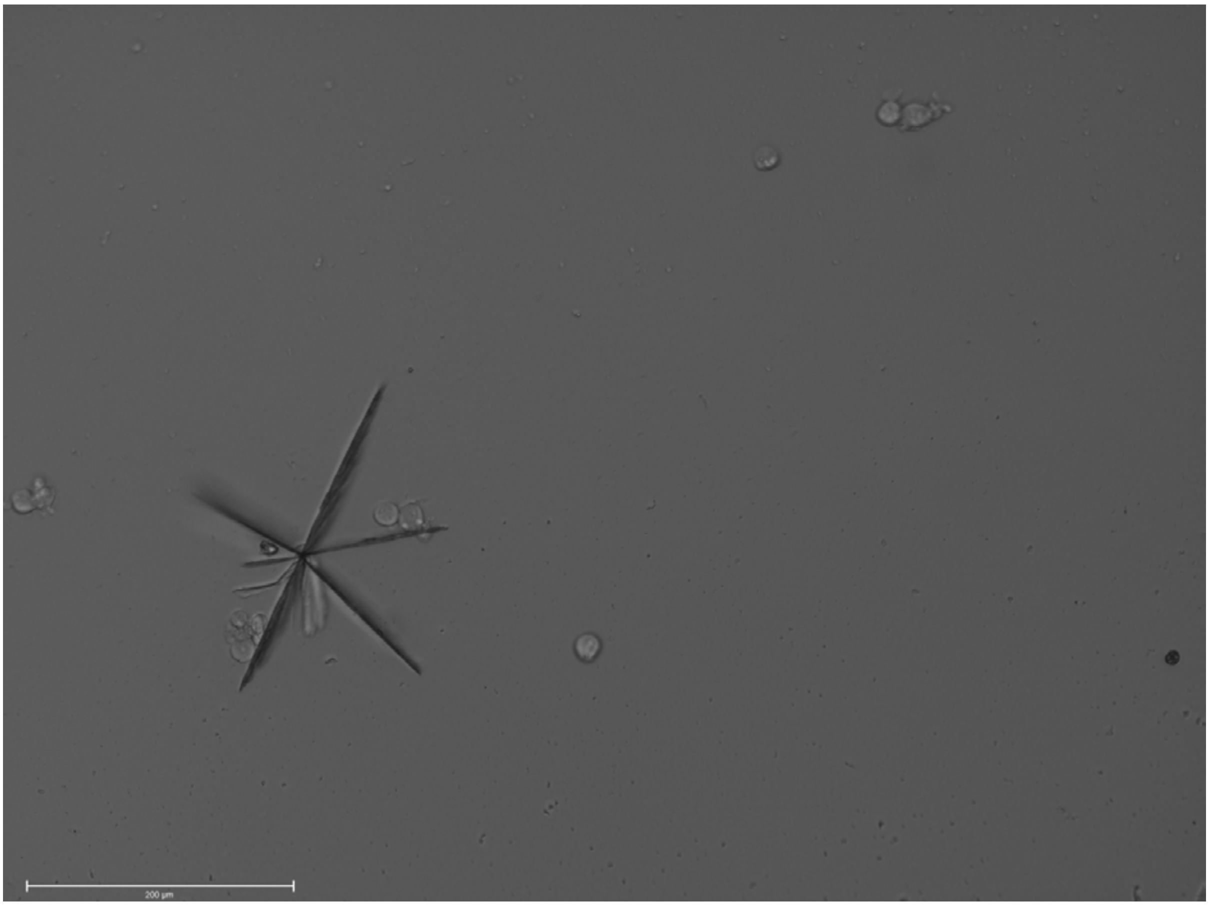

Neuro2a Type C

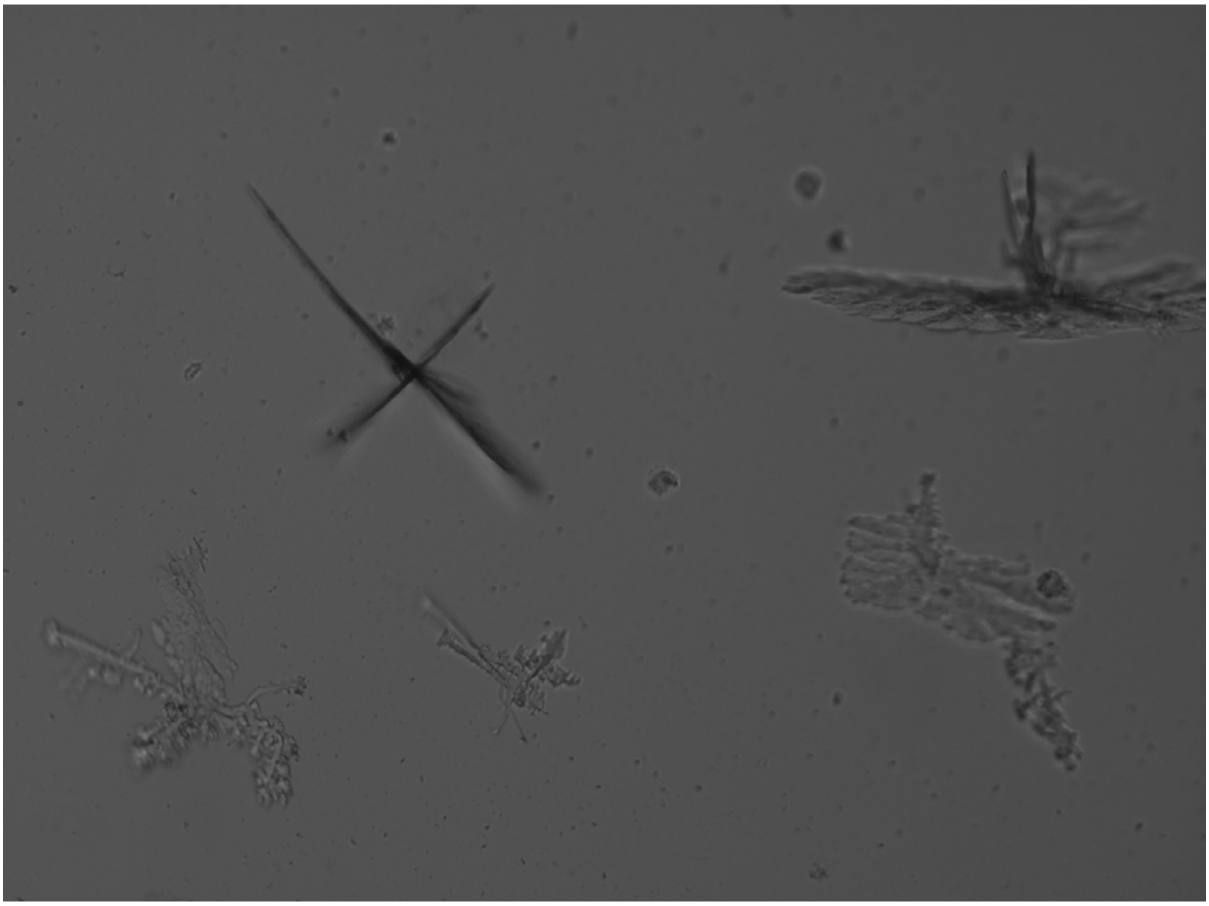

Neuro2a Type C

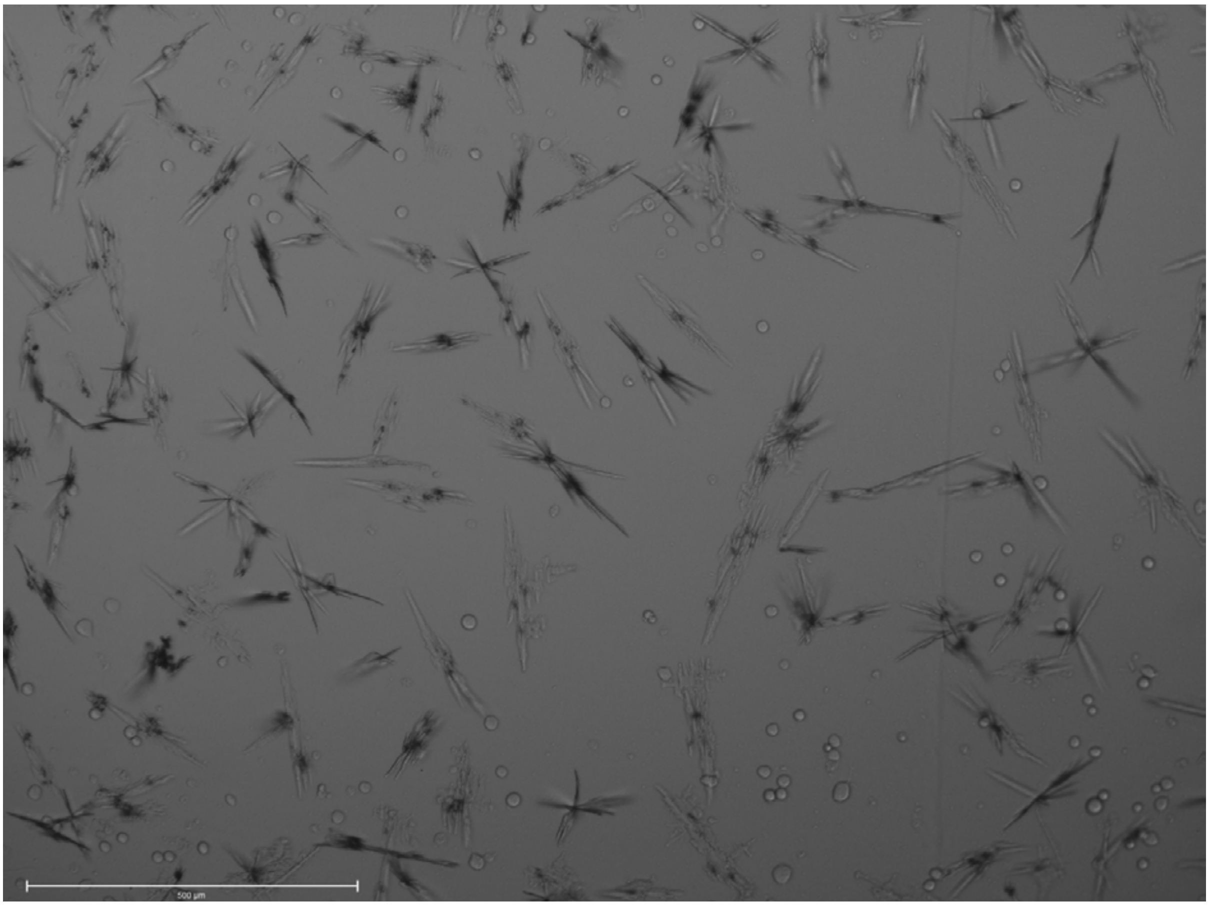

IMR-32 Control

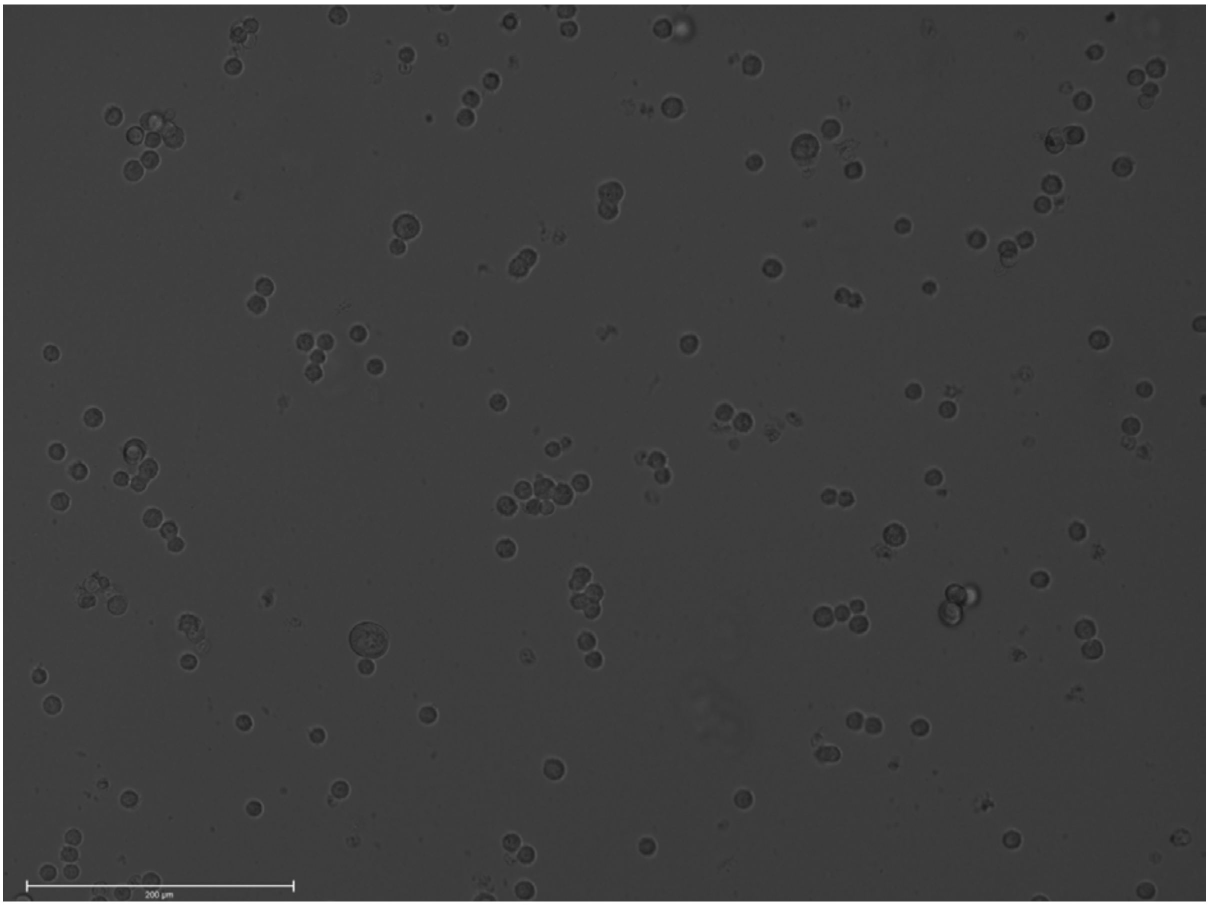

IMR-32 Control

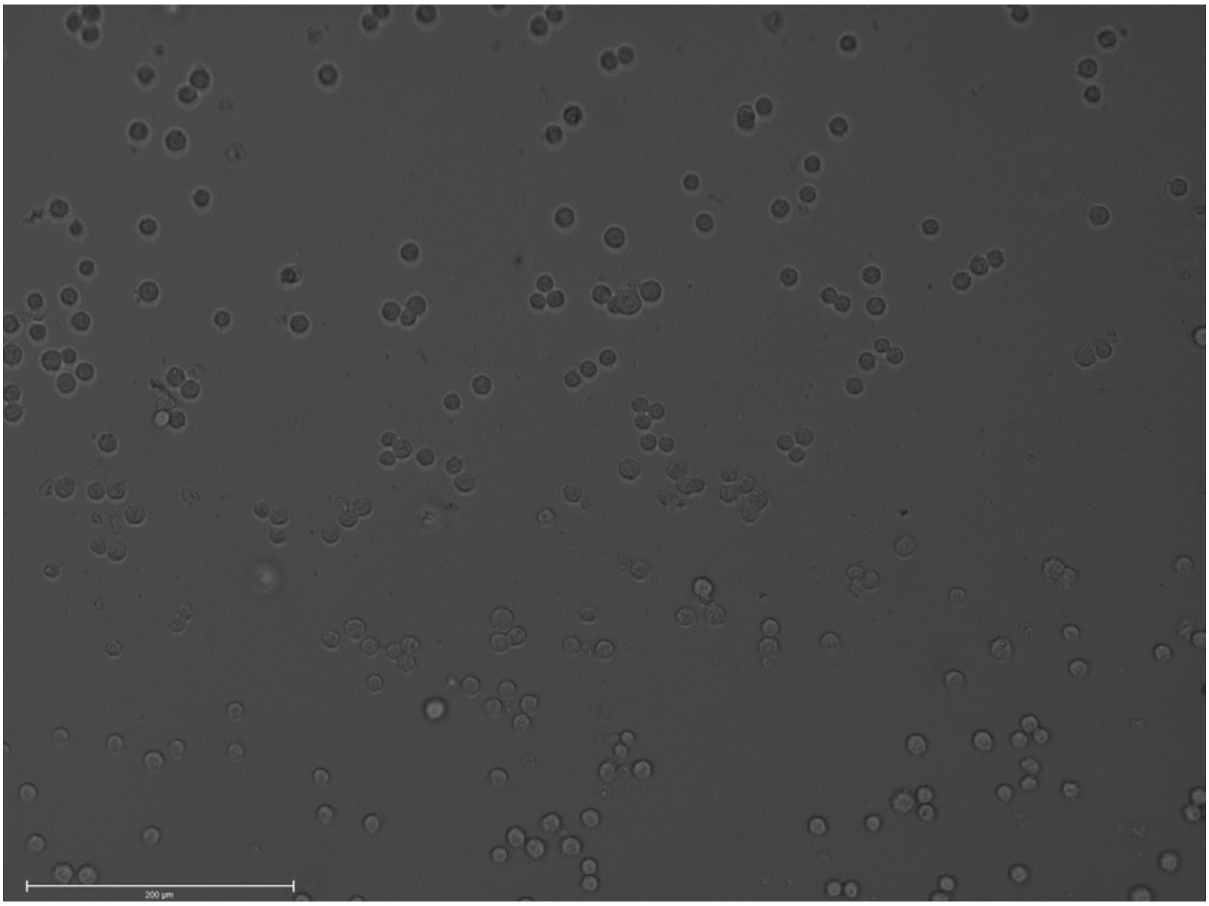

IMR-32 + 100µM FPRa14

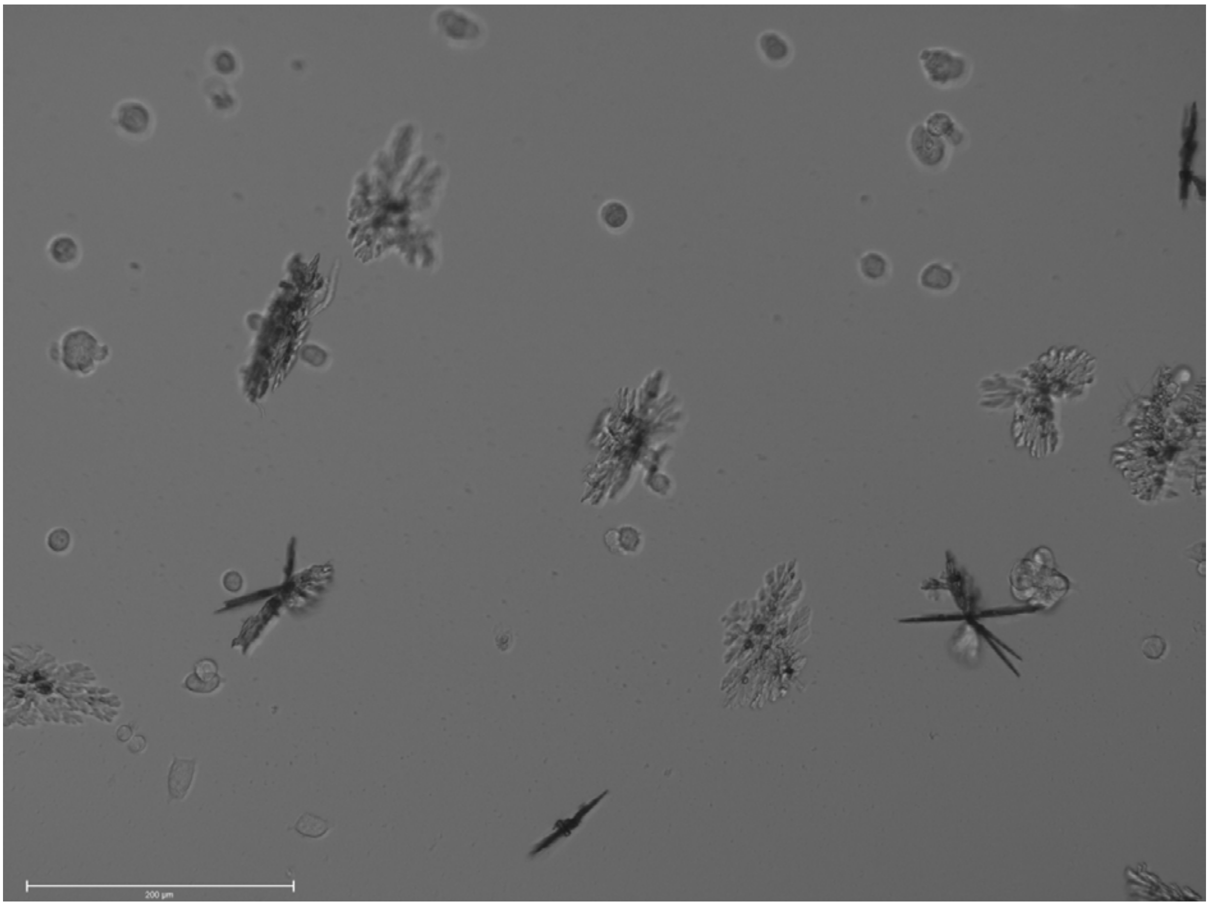

IMR-32 + 100µM FPRa14

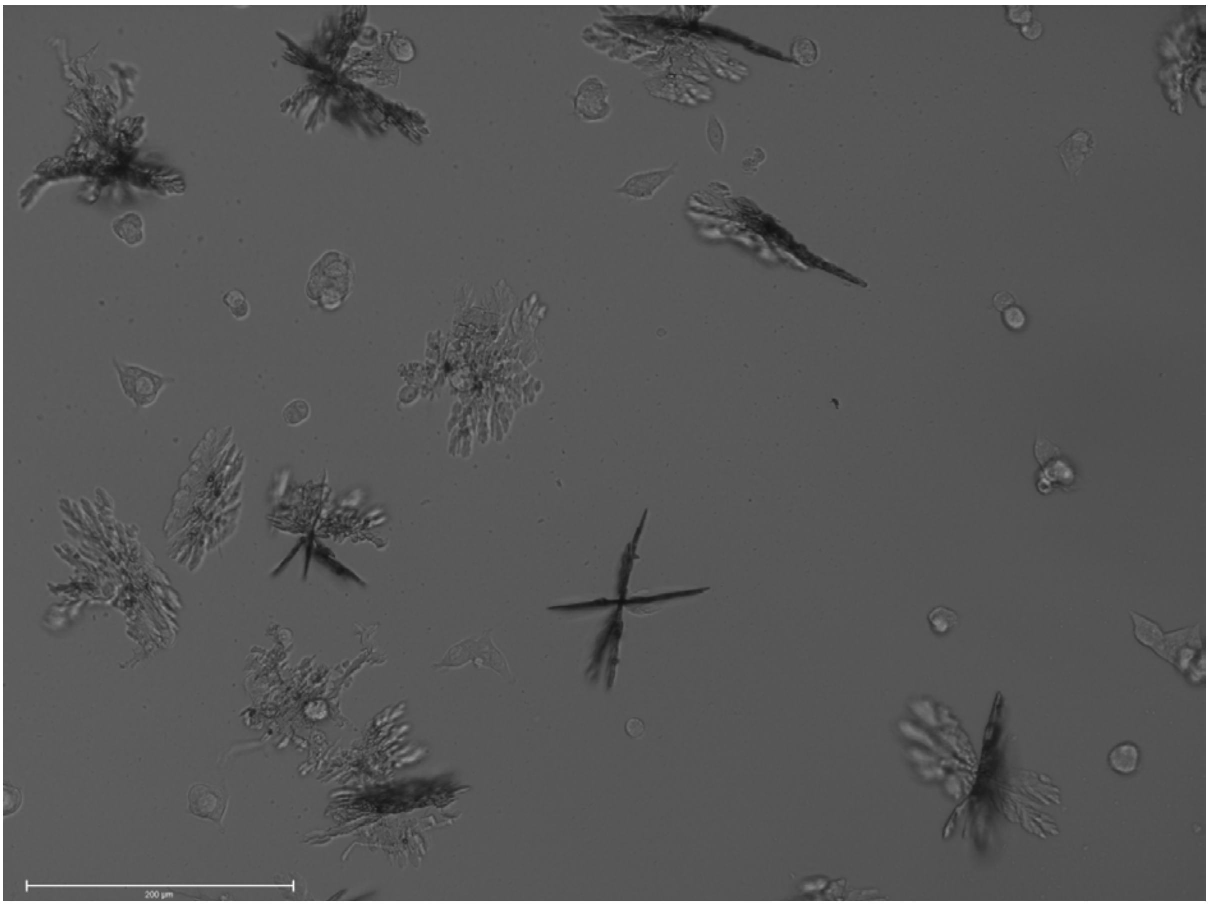

IMR-32 + 100µM FPRa14

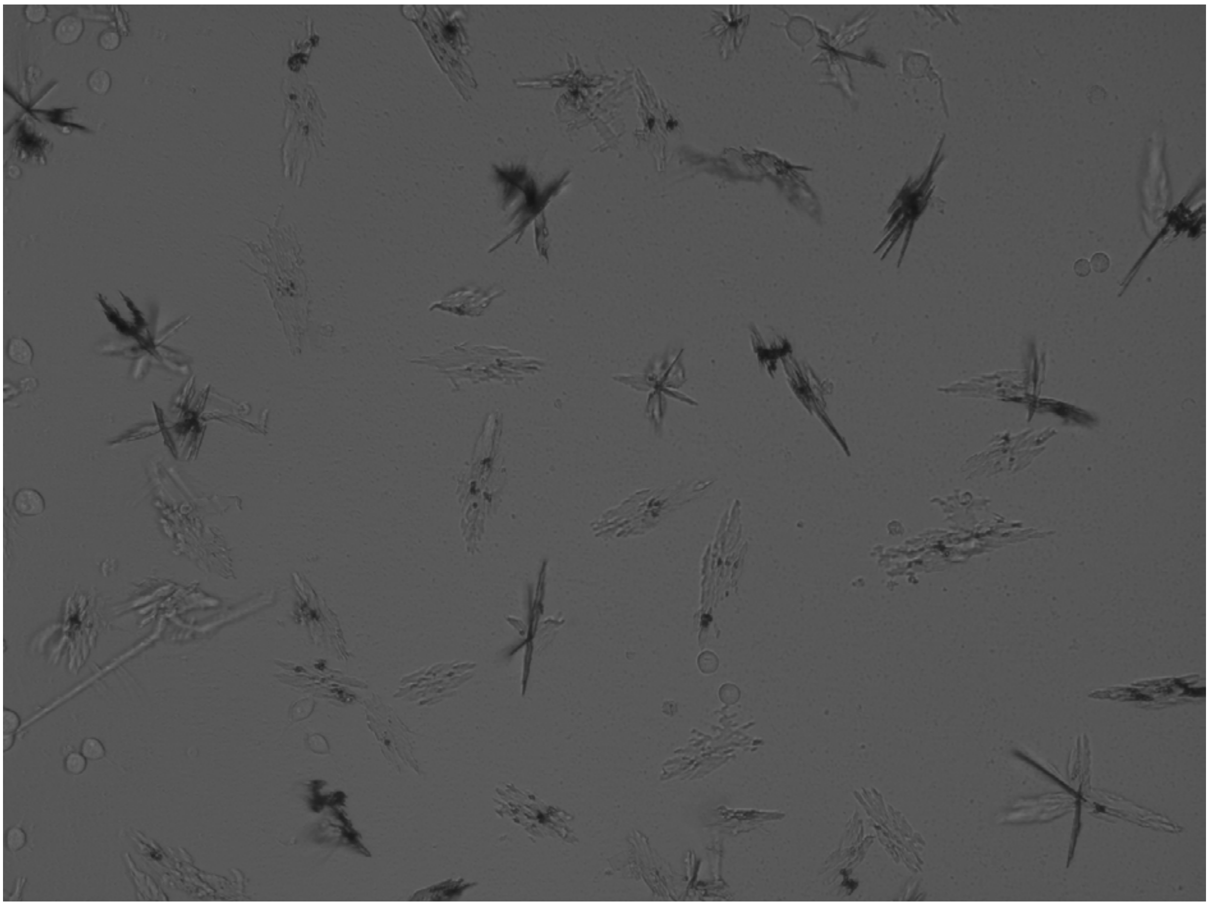

IMR-32 + 100µM FPRa14

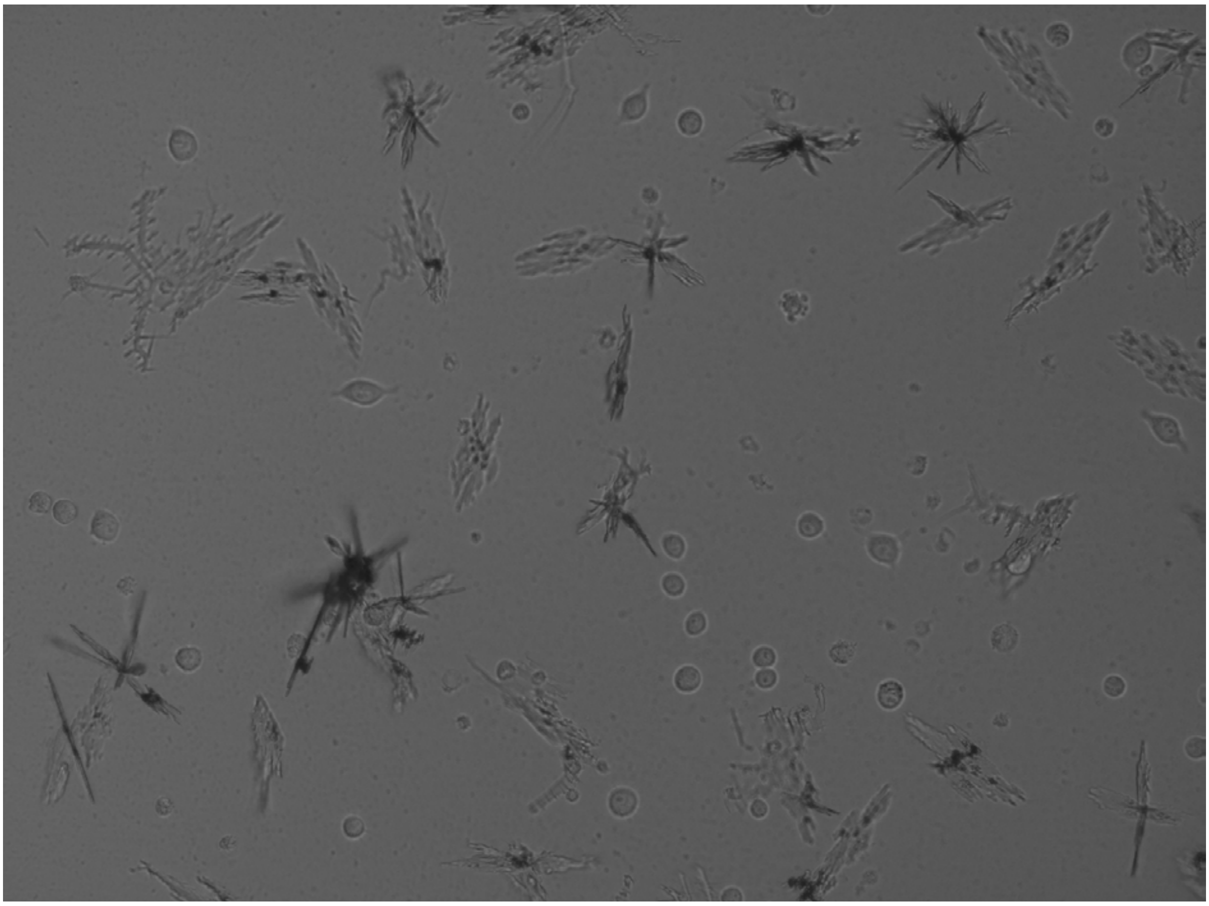

SH-SY5Y Control

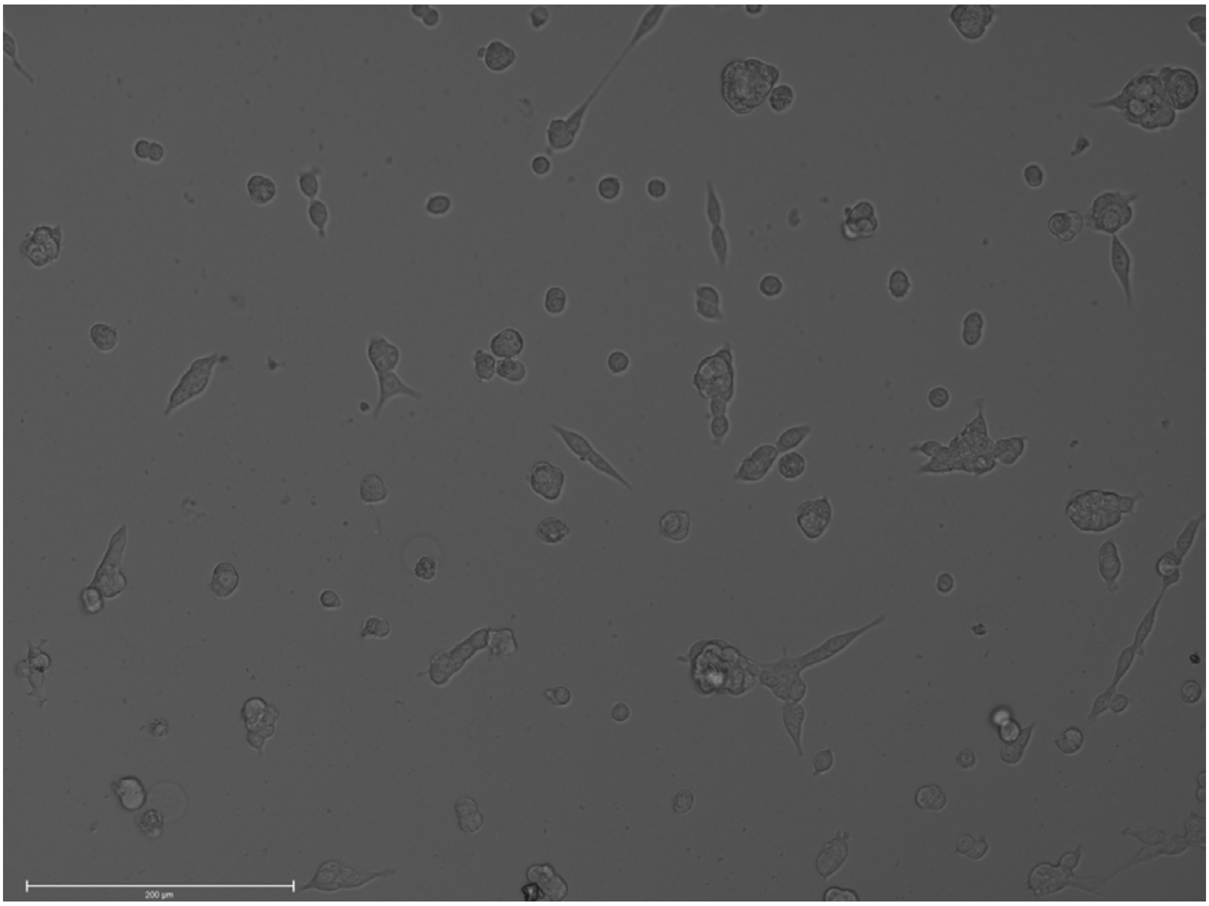

SH-SY5Y Control

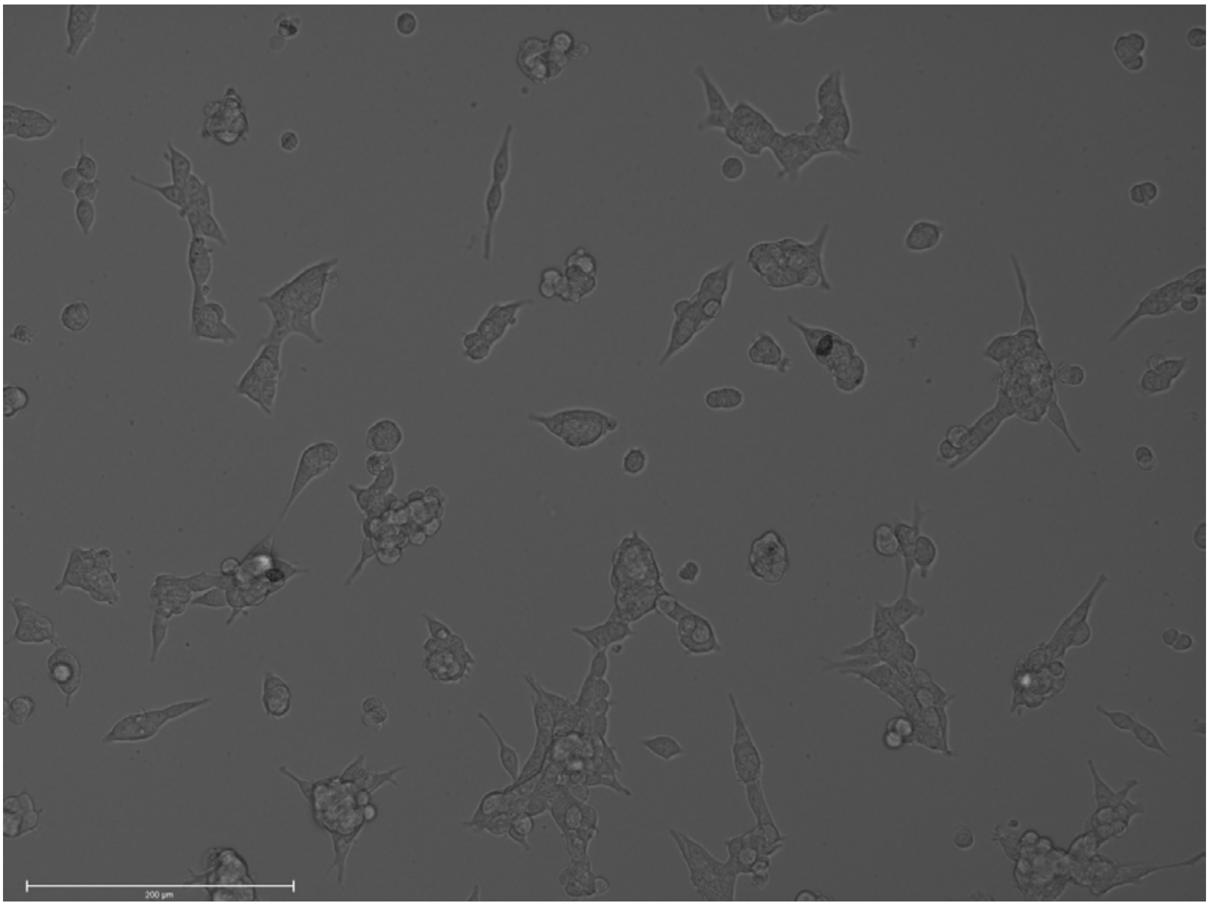

SH-SY5Y + 100µM FPRa14

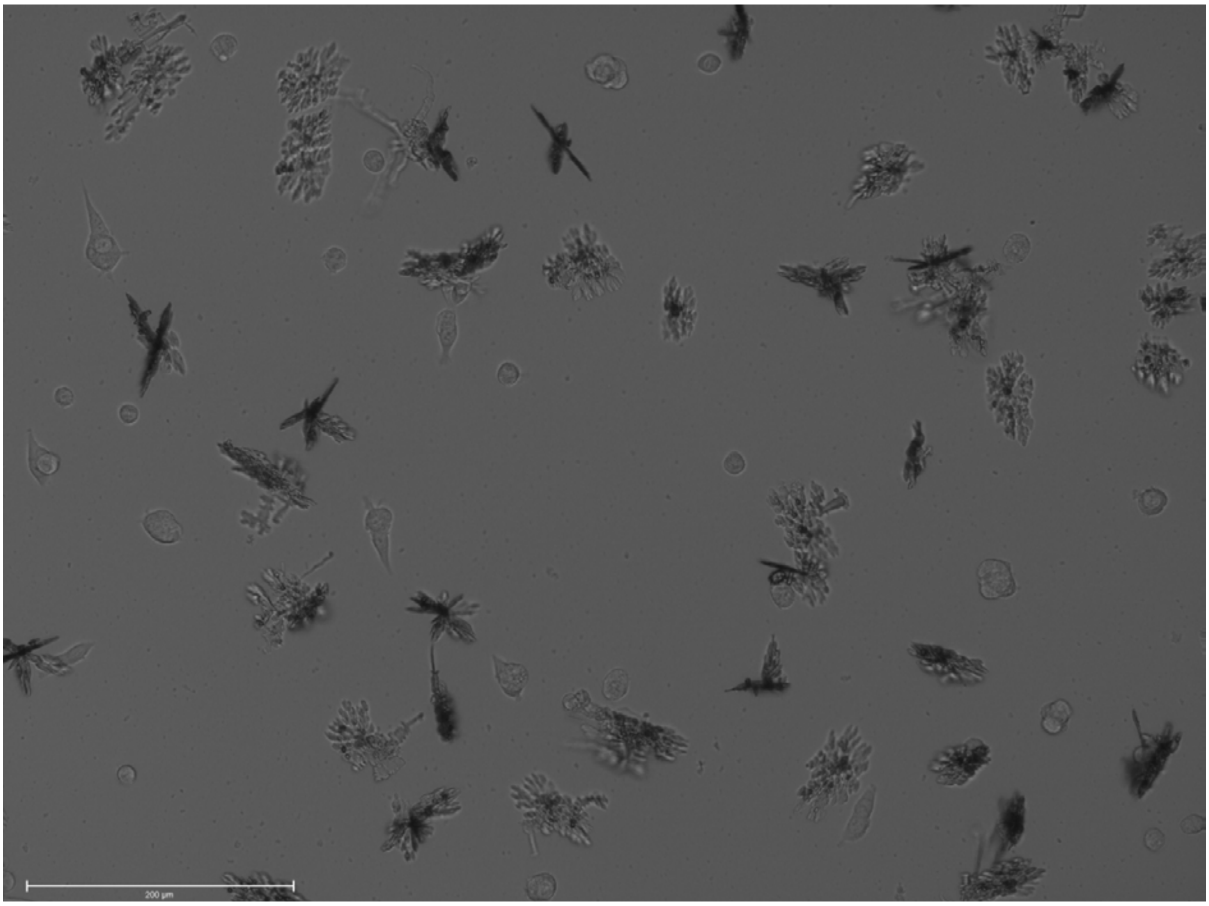

SH-SY5Y + 100μM FPRa14

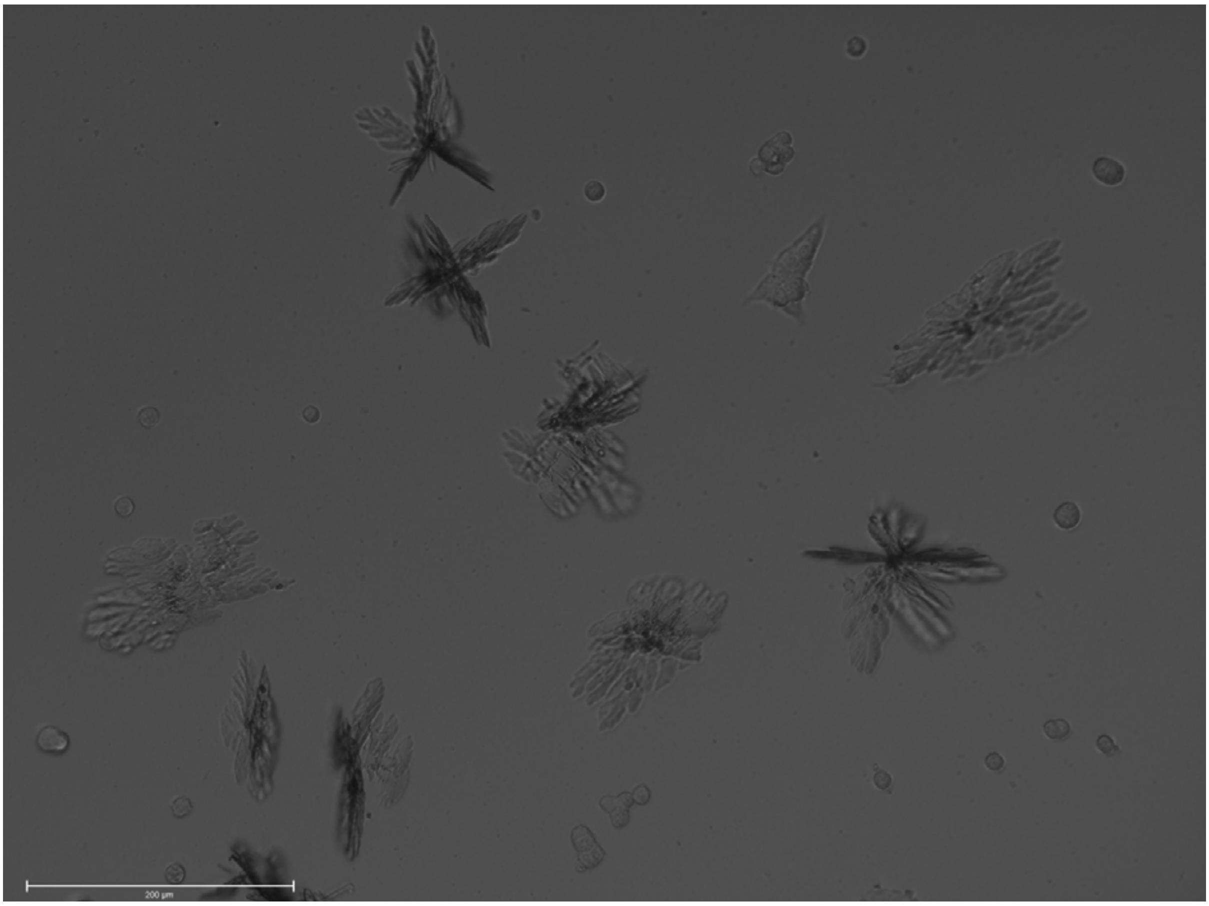

SH-SY5Y + 100µM FPRa14

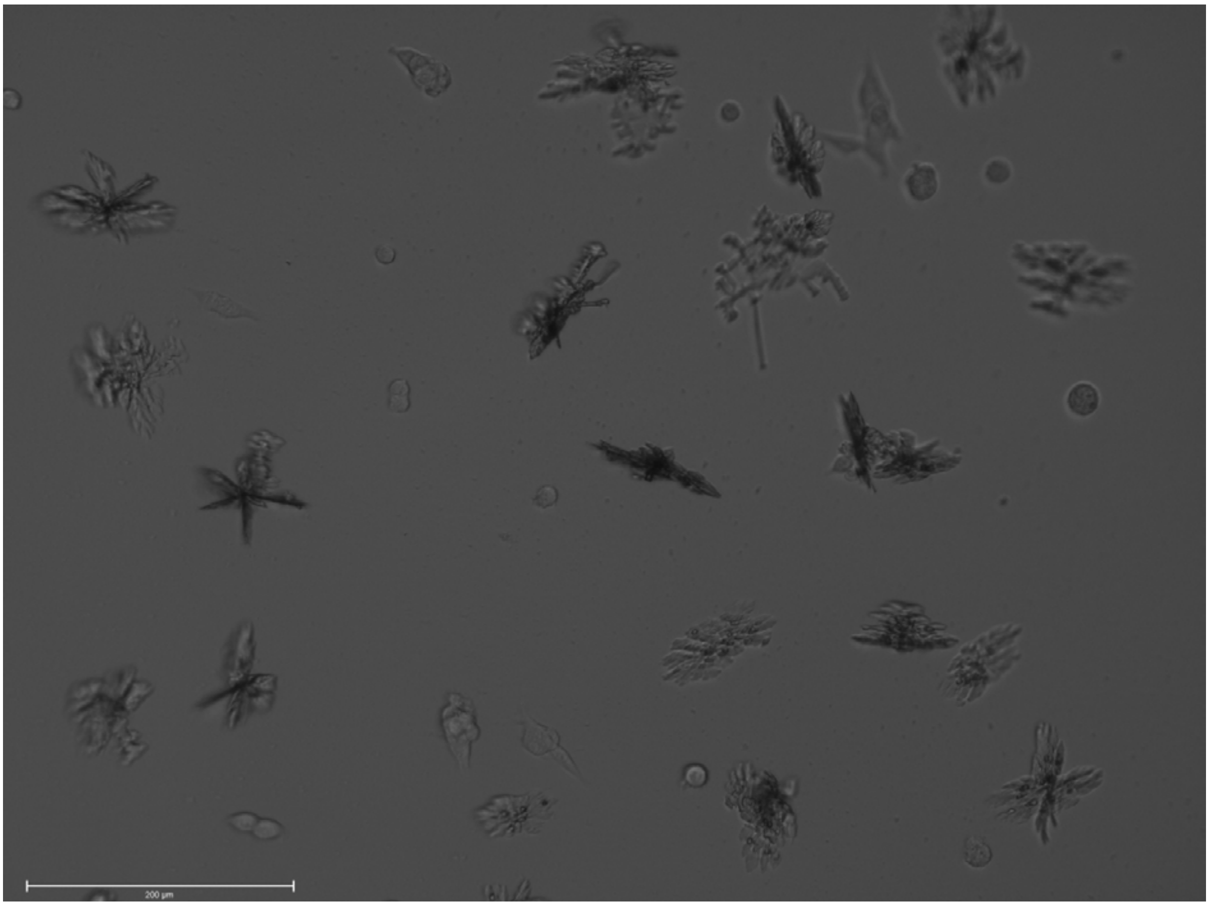

SH-SY5Y + 100µM FPRa14

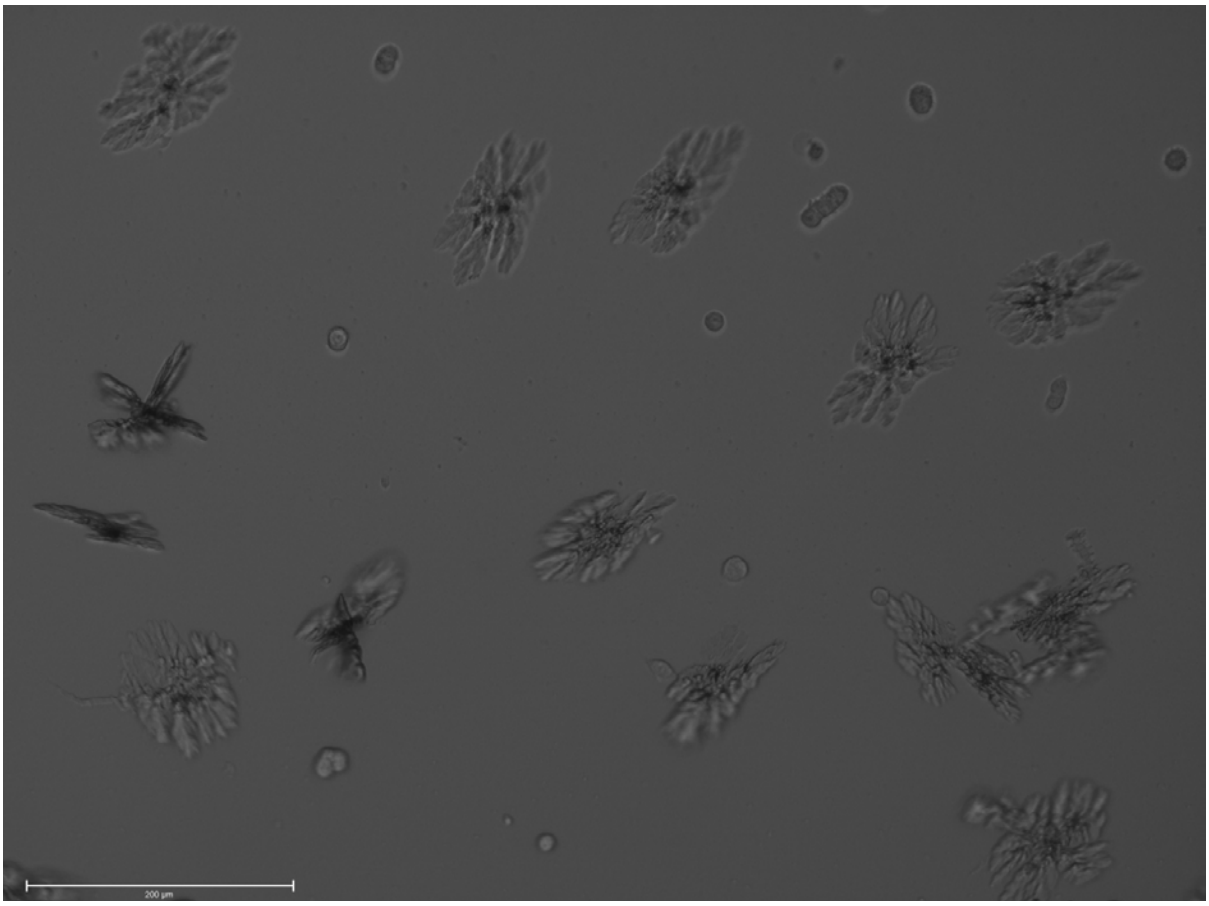

Supplement: S1 Figs — (PDF) [file pone.0217815.s001.pdf]
